# Microglial CX3CR1 deficiency regulates the selective vulnerability of cone photoreceptors via STAT3/CCL–ACKR1 signaling in the mouse retina

**DOI:** 10.1038/s12276-025-01618-7

**Published:** 2026-01-15

**Authors:** Rong Li, Jing Zhang, Qiong Wang, Jun-Qi Fan, Bin Lin

**Affiliations:** 1 School of Optometry, The Hong Kong Polytechnic University, Hong Kong, China; 2 Research Centre for SHARP Vision, The Hong Kong Polytechnic University, Hong Kong, China

**Keywords:** Cell death in the nervous system, Cell death and immune response, Chemokines, Chemokines, Retina

## Abstract

Selective neuronal vulnerability is a common feature of neurodegenerative disorders. However, the molecular mechanisms that drive this selective vulnerability are not fully understood. Here we observed that microglial CX3CR1 interference induced proinflammatory responses in microglia and astrocytes that were correlated with the selective vulnerability of cone photoreceptors in the mouse retina. Via proteomic analysis, we identified STAT3 as a potential downstream target by which CX3CR1 mediates microglial neurotoxicity. Moreover, single-cell RNA sequencing analysis revealed that CX3CR1-deficient microglia exhibit eight distinct transcriptomic phenotypes. At the mechanistic level, our data revealed that the involvement of *Tnf*-dominant microglia occurred mainly via microglia‒cone interactions through CCLs and their receptor, atypical chemokine receptor 1 (*Ackr1*), whose expression was upregulated primarily in cones through NF-κB signaling, leading to selective cone loss. In addition, we found that *Cxcl1*-dominant microglia primarily communicated with astrocytes via the *Bmp2*–*Bmpr1a*/*Bmpr1b* pair, leading to increased STAT3 levels and, consequently, elevated CCL and CXCL production in astrocytes, which in turn contributed to further cone loss through *Ackr1*. Overall, our data demonstrate that microglial CX3CR1 deficiency induces selective cone cell death via activation of the STAT3/CCL–ACKR1 signaling pathway, and that targeting CX3CR1/STAT3 could represent a therapeutic strategy to reduce microglial neurotoxicity.

## Microglial CX3CR1 deficiency drives cone cell death

This study explores why certain neurons in the brain are more vulnerable in degenerative contexts such as Alzheimer’s disease. The study investigates the role of CX3CL1/CX3CR1 signaling in selective neuronal vulnerability in the retina, an extension of the brain. They found that CX3CR1 deficiency in microglia activates STAT3 signaling, triggering proinflammatory responses in both microglia and astrocytes. This neuroinflammation, mediated by chemokines such as CCL and CXCL, specifically targets cone photoreceptors primarily expressing Ackr1, leading to their selective vulnerability and loss. These findings demonstrate that the CX3CR1/STAT3 pathway is a key mechanism driving selective neuronal damage, suggesting it as a promising therapeutic target for mitigating microglia-mediated neurotoxicity in neurological disorders.

This summary was initially drafted using artificial intelligence, then revised and fact-checked by the author.

## Introduction

Selective neuronal vulnerability is a common clinical manifestation of different neurodegenerative diseases, including Alzheimer’s disease (AD) and Parkinson’s disease^1–3^. Advances in single-cell sequencing have revealed particularly vulnerable and resilient neuronal subtypes in postmortem brains from patients with AD^1,4^. Understanding selective vulnerability would not only elucidate the key molecular mechanisms that drive neurodegeneration but also pave the way for more effective therapeutic strategies to increase resilience against neurodegenerative disorders. However, the mechanisms underlying this selective neuronal vulnerability have remained elusive.

Microglia, which are the resident macrophages of the brain, not only play important roles in brain homeostasis, including maintaining neural integrity, but also are implicated in diverse neurological conditions. C-X3-C motif chemokine receptor 1 (CX3CR1) is a chemokine receptor that is expressed on microglia and binds its only ligand, neuronally derived fractalkine (CX3CL1), to facilitate communication between microglia and neurons^5–8^; CX3CR1 plays an important role in maintaining homeostasis and regulating neuroinflammation and neuronal death in the brain^9–11^. Disruption of CX3CR1 is associated with increased neuronal loss and cognitive impairment in various neurodegenerative diseases^5,6,12,13^, suggesting the critical role of microglial CX3CR1 in mediating neurotoxicity. Similarly, CX3CR1 deficiency reportedly causes the loss of cone photoreceptor function during early postnatal development in mouse retinas^14^. In addition, we and others previously reported that genetic ablation of CX3CR1 accelerates cone photoreceptor dysfunction and loss in mouse models of inherited retinal degenerative diseases^14,15^, whereas delivery of CX3CL1 via gene therapy abrogates cone photoreceptor loss and ameliorates visual function in these mouse models^16^; these findings highlight the importance of CX3CL1/CX3CR1 signaling in retinal development and diseases. However, the involvement of CX3CL1/CX3CR1 signaling in selective neuronal vulnerability and the subsequent molecular mechanism underlying this selective neuronal vulnerability are still unknown.

Here, we investigated the changes in the cellular morphology and visual function of CX3CR1-deficient mice and the underlying mechanisms by which microglial CX3CR1 deficiency induces neurotoxicity through a series of in vivo and in vitro studies involving a combination of molecular biology, proteomic and transcriptomic analyses. Our data revealed that dysregulation of CX3CR1 signaling activated microglia, resulting in increased proinflammatory responses in microglia and then astrocytes and subsequently inducing toxicity associated with the selective vulnerability of cone photoreceptors in CX3CR1-deficient retinas. Using proteomic analysis, we identified STAT3 as a potential downstream target by which CX3CR1 mediates microglial neurotoxicity. Subsequent single-cell RNA sequencing (scRNA-seq) analysis revealed that microglial CX3CR1 deficiency resulted in transcriptional heterogeneity in microglia. Furthermore, *Tnf*-dominant microglia communicate with cone photoreceptors mainly via chemokine CCL signals and their receptor, atypical chemokine receptor 1 (*Ackr1*); *Ackr1* expression was upregulated primarily in cone photoreceptors in the context of microglial CX3CR1 deficiency, and this upregulation subsequently increased the selective vulnerability of cone cells. Moreover, *Cxcl1*-dominant microglia are primarily responsible for interactions with astrocytes via *Bmp2*–*Bmpr1a*/*Bmpr1b* signaling, resulting in astrocyte reactivity and increased production of CCL and CXCL, which function with *Ackr1* in cone cells, contributing to increased cone cell loss. Together, our findings demonstrate that CX3CR1/STAT3 signaling might be an important mediator of selective neuronal vulnerability and that targeting CX3CR1/STAT3 signaling could be a promising therapeutic approach for eliminating microglial neurotoxicity in neurological disorders.

## Materials and methods

### In vivo experiments

#### Mice

C57BL/6J (stock no. 000664) and CX3CR1^GFP/GFP^ (stock no. 005582) mice were obtained from the Jackson Laboratory. C57BL/6J mice were backcrossed with CX3CR1^GFP/GFP^ mice to generate CX3CR1^+/GFP^ mice on C57BL/6J background, and the littermates from CX3CR1^+/GFP^/C57BL/6J were used for flow cytometry analysis. All mice were housed in a 12-h light/dark cycle with water and food ad libitum, and maintained at the Centralised Animal Facilities, The Hong Kong Polytechnic University. All experimental procedures were approved by the Animal Subjects Ethics Sub-committee (ASESC) of The Hong Kong Polytechnic University and conducted in accordance with the Association for Research in Vision and Ophthalmology (ARVO) statement for the use of animals. A total of approximately 60 CX3CR1^GFP/GFP^ and 40 C57BL/6J mice (6 weeks of age) were used in this study.

#### Intravitreal siRNA injections

To study the role of STAT3 on glial reaction and selective cone photoreceptor loss in retinas of CX3CR1^GFP/GFP^ mice, we injected intravitreally the STAT3 specific small interfering RNA (siRNA; Thermo Fisher Scientific) or AAV9-packaged siSTAT3 with microglia-specific promoter F4/80p^17,18^ (GeneChem) into CX3CR1^GFP/GFP^ mouse eyes at 6 weeks of age as previously described^19,20^. In brief, mice were anesthetized with a mixture of ketamine hydrochloride (100 mg/kg) and xylazine (50 mg/kg) and then placed under a dissecting microscope. After making an incision in the superior nasal sclera using a sterile and sharp 31 G needle, a glass pipette carrying 1 µl siRNA solution was carefully inserted into the same incision and slowly injected into the vitreous. After injections, antibiotic eye ointment was applied to prevent infection. A total of 100 µM STAT3 siRNA or negative control siRNA was intravitreally injected to CX3CR1^GFP/GFP^ mouse eyes four times, two days apart. The knockdown efficiency of STAT3 was evaluated by quantitative polymerase chain reaction (qPCR) and western blot. Then, 1 × 10^13^ vg/ml AAV-F4/80p-siSTTA3-mCherry or AAV-F4/80p-siCTR-mCherry was intravitreally injected to CX3CR1^GFP/GFP^ mouse eyes one time, and retinas were collected for further analysis after 4 weeks. The transfection efficiency was evaluated by colocalization of mCherry^+^ and GFP^+^ cells.

#### Colivelin TFA treatment

The role of STAT3 signal elevation was investigated by pharmacological treatment with the STAT3-specific activator Colivelin trifluoroacetate (TFA) (MedChemExpress) in the retina of CX3CR1^GFP/GFP^ mice at 6 weeks of age. Colivelin TFA was dissolved in phosphate-buffered saline (PBS) and administrated intraperitoneally at a dose of 1 mg/kg into the CX3CR1^GFP/GFP^ mice for 14 consecutive days. To investigate the causal role of STAT3 signaling in microglial neurotoxicity, CX3CR1^GFP/GFP^ mice were initially administered with AAV-F4/80p-siSTAT3-mCherry or AAV-F4/80p-siCTR-mCherry. After 14-day treatments, these mice were treated with Colivelin TFA for another 14 consecutive days before collecting retina samples for further analysis.

#### Immunocytochemistry and confocal imaging

After enucleation, the retina was separated from the vitreous and sclera in PBS and fixed in 4% paraformaldehyde for 1 h, followed by dehydration in 30% sucrose overnight at 4 °C. Some of the retinas were embedded in the Tissue-Tek O.C.T. Compound (OCT) and underwent serial frozen sectioning at a thickness of 14 µm on a cryostat microtome. After rinsing three times with PBS, the retina sections were incubated with rat CD68 (Bio-Rad, 1:500), rabbit GFAP (Dako, 1:500), rabbit R/G opsin (Millipore, 1:250) and Caspase-3 (Cell Signaling Technologies, 1:200) in blocking buffer containing 3% normal donkey serum, 1% bovine serum albumin (BSA) and 0.3% Triton X-100 in PBS, pH 7.4, overnight. Afterwards, donkey anti-rabbit Alexa Flour 594 (Invitrogen, 1:500) and donkey anti-rat Alexa Fluor 555 (Abcam, 1:500) or lectin peanut agglutinin (PNA) (Vector Labs, 1:100) were incubated for 2 h before mounting slides with Dako fluorescence mounting medium. For whole-mounted retinas, CD68 (Bio-Rad, 1:500) and R/G opsin (Millipore, 1:250) were individually incubated for 24 h and 72 h, followed by incubating with donkey anti-rat Alexa Fluor 555 (Abcam, 1:500) and donkey anti-rabbit Alexa Flour 594 (Invitrogen, 1:500) for 2 h. Fluorescence images of retinal sections and whole-mounted retinas were captured by a Zeiss LSM 800 Upright Confocal Microscope (Zeiss) with a pixel resolution of 1,024 × 1,024 and Plan-Apochromat 20×/0.8 objective. *Z*-stack images with an interval of 0.8 µm was acquired. For the quantification of fluorescence intensity of GFAP and the number of R/G opsin-positive cone photoreceptors, three areas at 100 µm (central), 1 mm (middle) and 1.8 mm (peripheral) from the optical nerve head in each retinal section were captured. The mean immunofluorescence intensity of GFAP was calculated with ImageJ software, as previously described^20,21^. The number of R/G opsin-positive or PNA-positive cone photoreceptors in each capture was manually counted. For quantification of microglial cells, four sampling areas with 638.9 µm × 638.9 µm squares along the dorsal–ventral axis of retinal whole mounts at 200 µm and 1 mm from the optic nerve head on both sides were photographed, and the numbers of GFP^+^ and of CD68^+^GFP^+^ microglial cells were manually counted.

#### Immunoblotting

Animals were anesthetized with a mixture of ketamine hydrochloride (100 mg/kg) and xylazine (50 mg/kg), and eyeballs were quickly enucleated, followed by separating the retina from vitreous and sclera in PBS. Total proteins were extracted with RIPA buffer (Abcam) containing proteinase and phosphatase inhibitor cocktails (Roche) on ice for 30 min. After centrifugation at 10,000*g* for 15 min, the supernatant was collected, and protein concentration was quantified with Pierce rapid gold BCA protein assay kit (Invitrogen). Western blot was performed as previously described^20^. In brief, 20 μg of protein was loaded into 10% SDS–PAGE gel. After running the gel at 80 V for 90 min, the proteins in the gel were transferred to a polyvinylidene difluoride membrane. Primary antibodies including rabbit p-STAT3 (Cell Signaling Technology, 1:1,000), rabbit STAT3 (Cell Signaling Technology, 1:1,000), mouse GAPDH (Millipore, 1:2,000) and mouse β-actin (Invitrogen, 1:2,000) were incubated overnight at 4 °C after blocking the membrane containing proteins with 5% BSA for 1 h. Afterwards, the membrane was incubated with goat anti-rabbit IgG and goat anti-mouse IgG (Invitrogen, 1:1,000) conjugated to horseradish peroxidase for 2 h. The membrane with protein was evaluated by ChemiDoc Imaging Systems (Bio-Rad) after incubating with SuperSignal West Pico PLUS Chemiluminescent Substrate (Invitrogen) or ECL Select Western Blotting Detection Reagent (Amersham). The optical density value of each band was measured using ImageJ software.

#### Quantitative real-time PCR

Total RNA from retina tissues was extracted with the TransZol Up Plus RNA Kit (TransGen) or TRIzol reagent (Invitrogen), and cDNA was synthetized using the TransScript First-Strand cDNA Synthesis SuperMix (TransGen) or PrimeScript RT Master Mix (TaKaRa). qPCR was performed with PerfectStart Green qPCR SuperMix (TransGen) or TB Green Premix Ex Taq (Tli RNase H Plus) (TaKaRa) using the QuantStudio 7 Flex Real-Time PCR System (Applied Biosystems) or QuantStudio 5 Flex Real-Time PCR System (Applied Biosystems). GAPDH was used as a control, and the data were analyzed using the 2^−^^ΔΔ^^Ct^ method. Gene-specific primers used for qPCR are listed in Supplementary Table 1.

#### Electroretinogram (ERG) analysis

ERGs were performed as previously described by us^22^. In brief, mice were anesthetized with a mixture of ketamine hydrochloride (100 mg/kg) and xylazine (50 mg/kg) after dark adaption overnight, followed by placing the mice on a platform heater at 37 °C and applying 3% Hypromellose lubricating gel solution in both corneas and the cups of the electrodes. ERG was performed using a Celeris ERG system (Diagnosys) with the TOUCH/TOUCH protocol. A scotopic ERG was detected at 0.01, 0.1, 1 and 3 cd·s/m^2^ light intensities, followed by photopic ERG detection at 3 and 10 cd·s/m^2^ light intensities after 10-min light adaptation with background light intensity at 30 cd·s/m^2^. Ten sweeps were acquired with each light stimulus. The amplitude of the ERG a-wave was measured from the baseline to its negative peak, and the amplitude of the b-wave was measured from the the bottom of the a-wave to the top of the tallest curve.

#### Mass spectrometry

Retinas from CX3CR1^GFP/GFP^ and C57BL/6J mice at 6 weeks of age were collected, and five samples in each group were applied for proteomics analysis. The total proteins were extracted with a cocktail lysis buffer containing SDS L3, EDTA and dithiothreitol on ice for 15 min and quantified with the Bradford quantification assay (Invitrogen). In total, 100 µg of proteins were digested with sequencing-grade trypsin in 50 mM NH_4_HCO_3_ solution for 4 h at 37 °C, followed by desalting with a Strata X column and vacuum drying. The dried peptides were reconstituted with mobile phase A (2% acetonitrile, 0.1% formic acid) and subjected to a Thermo UltiMate 3000 UHPLC liquid chromatograph coupling a tandem self-packed C18 column for separation at a flow rate of 500 nl/min by the following effective gradient: ~0–5 min, 5% mobile phase B (98% acetonitrile, 0.1% formic acid); ~5–90 min, mobile phase B linearly increased from 5% to 25%; ~90–100 min, mobile phase B rose from 25% to 35%; ~100–108 min, mobile phase B rose from 35% to 80%; ~108–113 min, 80% mobile phase B; ~113.5–120 min, 5% mobile phase B. Next, the peptides separated by liquid phase chromatography were ionized by a nanoESI source and then passed to a tandem mass spectrometer Oritrap Exploris 480 (Thermo Fisher Scientific) for data-independent acquisition mode detection. The main parameters were set as follows:ion source voltage, 1.9 kV; MS1 scanning range, ~400–1,250 *m*/*z*; resolution, 120,000; maximum injection time, 90 ms; the 400–1,250 *m*/*z* range was divided into 50 continuous windows per MS/MS scan; MS/MS resolution, 30,000; and AGC targets of 300% for MS and 1000% for MS/MS. For bioinformatic analysis, all data-independent acquisition data underwent an analytical quality control with mProphet algorithm to obtain the reliable quantitative results. The raw MS data were processed with MaxQuant (version 2.0.1.0), and proteins were identified with the UniProt *Mus musculus* database. Differentially expressed proteins (DEPs) were screened with protein fold change (FC) ratio >1.5 or <1/1.5 above the 95% confidential level, and *P* value <0.05 in each comparison. Gene Ontology (GO) annotation and Kyoto Encyclopedia of Genes and Genomes (KEGG) pathway analysis for all DEPs were analyzed using the online AmiGo2 (http://amigo.geneontology.org/amigo) and KEGG pathway database (https://www.genome.jp/kegg/). Protein–protein interaction (PPI) analysis was performed with online STRING software (version 11.5).

#### Flow cytometry

After enucleation, retinas from CX3CR1^GFP/GFP^, CX3CR1^+/GFP^ and C57BL/6J mice at 6 weeks of age were quickly collected in PBS. Each retina was dissociated with 150 µl papain enzyme in EBSS solution and 12.5 µl DNase (Worthington Biochemical Corporation) at 37 °C for 30 min. After centrifugation at 300*g* for 5 min, the cell pellets were resuspended in 2% BSA for one wash, followed by fixing cells with Intracellular (IC) fixation buffer (Invitrogen) for 30 min at room temperature. Afterwards, cell samples were centrifugated and incubated with precooled 90–100% methanol for 40–60 min at 4 °C. After a single rinse, cell samples were blocked with Fc blocking antibody (Invitrogen) in cell staining buffer (BioLegend) for 10 min before incubating with mouse Phospho-STAT3 (Tyr705) monoclonal antibody (p-STAT3-APC, Invitrogen), mouse GFAP antibody (GFAP-PE, BD Bioscience) and rabbit R/G opsin antibody (Merk Millipore) for 30–40 min. For cone photoreceptor staining, after incubating with R/G opsin antibody, cell samples were stained with donkey anti-rabbit IgG (H + L) highly cross-adsorbed secondary antibody conjugated with Alexa Fluor 594 for 30 min. After centrifugation and wash, cell pellets were resuspended in cell staining buffer and analyzed with BD FACSAria III Cell Sorter (BD Bioscience). For the detection of p-STAT3^+^/TNF-α^+^, p-STAT3^+^/CD68^+^ and p-STAT3^+^/CXCL1^+^ microglia, cell suspensions from CX3CR1^GFP/GFP^ and C57BL/6J retinas were stained with CD11b-FITC (Invitrogen) and CD68-PE (Invitrogen) for 30 min on ice before fixation. After one wash, cells were incubated with mouse Phospho-STAT3 (Tyr705) monoclonal antibody (p-STAT3-APC, Invitrogen), rabbit TNF-α antibody (Cell Signaling Systems) and CXCL1 antibody (R&D Systems), followed by incubation with donkey anti-rabbit IgG (H + L) highly cross-adsorbed secondary antibody conjugated with Alexa Fluor 594 for 30 min. All data were analyzed with FowJo V10 software.

#### Magnetic-activated cell sorting (MACS)

Fresh retinas from CX3CR1^GFP/GFP^ and C57BL/6J mice at 6 weeks of age were collected in precooled Hank’s Balanced Salt Solution and dissociated with papain enzyme and DNase at 37 °C for 30 min. After centrifugation at 1500 rpm for 5 min, cell pellets were resuspended in MACS buffer for one wash. Afterwards, cells were incubated with Fc blocking antibody for 10 min on ice, followed by incubation with anti-CD11b magnetic microbeads (Miltenyi Biotec) or anti-ACSA-2 microbeads (Miltenyi Biotec) for 15–20 min at 4 °C. After one wash, cells were resuspended in MACS buffer and loaded into a prebalanced LS column (Miltenyi Biotec) with 3 ml MACS buffer, which had been placed in the magnetic separator (Miltenyi Biotec). The unlabeled flow-through containing unlabeled cells was discarded, and the LS column containing labeled cells was washed with 6–8 ml MACS buffer before removing the LS column from the separator and quickly flushing out magnetically labeled cells into a 15-ml Falcon tube. The labeled cells were collected after centrifugation for further analysis. For p-STAT3 staining, MACS-sorted microglia or astrocytes were seeded in a sterile 12-well plate and incubated with culture medium containing 10% fetal bovine serum, 1% penicillin–streptomycin and GlutaMAX supplement or N-2 supplement individually for 24 h, followed by changing medium. On the seventh day^23,24^, the cells were collected for immunofluorescence analysis.

#### Enzyme-linked immunosorbent assay (ELISA)

For the detection of TNF-α, CCL2, CCL3, CCL4 and CXCL12 expressions in cell supernatants from MACS-sorted microglia or astrocytes in CX3CR1^GFP/GFP^ and C57BL/6J retinas at 6 weeks of age, TNF alpha Mouse Uncoated ELISA Kit (Invitrogen), MCP-1/CCL2 Mouse Uncoated ELISA Kit (Invitrogen), Mouse CCL3/MIP-1 alpha Quantikine ELISA Kit (R&D system), Mouse CCL4/MIP-1 beta DuoSet ELISA (R&D system) and SDF-1 alpha/CXCL12 Mouse ELISA Kit (Invitrogen) were applied. In brief, MACS-sorted microglia or astrocytes from CX3CR1^GFP/GFP^ and C57BL/6J retinas were seeded in a sterile 12-well plate and incubated with culture medium for 7 days before collecting cell supernatants from primary culture microglia (pMCM) and primary culture astrocytes (pACM) for further analysis. Cellular products TNF-α, CCL2, CCL3, CCL4 and CXCL12 were quantified according to the corresponding manuals.

### scRNA-seq

#### Sample preparation, library construction and reads filtering for scRNA-seq

For scRNA-seq on microglia, retinas from CX3CR1^GFP/GFP^ and C57BL/6J mice at 6 weeks of age (*n* = 18–20 mice per group) were dissociated individually with papain dissociation system (Worthington Biochemical Corporation) for 30 min at 37 °C. After centrifugation at 400*g*, 4 °C for 5 min, cell pellets were resuspended in 2% BSA for one wash followed by blocking cell samples with Fc blocking antibody (Invitrogen) on ice for 10–15 min. Afterwards, cell samples from C57BL/6J mice were incubated with fluorescent isothiocyanate (FITC)-conjugated anti-CD45 (Invitrogen) and phycoerythrin (PE)-conjugated anti-CD11b (Invitrogen) in staining buffer for 30 min on ice. After rinsing one time, cell samples were analyzed with BD FACSAria III Cell Sorter (BD Biosccence). CD11b^+^/CD45^+^ cells^25^ from C57BL/6J or GFP^+^ cells from CX3CR1^GFP/GFP^ were sorted as microglia, and microglia with >85% cell viability were prepared for scRNA-seq. For scRNA-seq on whole retinal cells, single-cell suspensions from CX3CR1^GFP/GFP^ and C57BL/6J retinas at 6 weeks of age (*n* = 6 mice per group) were prepared as described above. After one wash, cell pellets were resuspended in 2% BSA. After assessing cell viability, qualified whole retinal cells were prepared for scRNA-seq analysis.

The single-cell libraries were constructed with the 10x Genomics platform, and the sequencing was performed using the DNBSEQ sequencing platform. The sequencing depth^26,27^ for microglial cells in the CX3CR1^GFP/GFP^ and C57BL/6J groups was 81.6k and 297.9k mean reads per cell, respectively, and the sequencing depth for whole retinal cells in the CX3CR1^GFP/GFP^ and C57BL/6J groups was 18.6k and 14.7k mean reads per cell, respectively. The sequencing saturation rates for microglial cells in the CX3CR1^GFP/GFP^ and C57BL/6J groups were 84.8% and 91.5%, and the sequencing saturation rates for whole retinal cells in the CX3CR1^GFP/GFP^ and C57BL/6J groups were 54.9% and 49.7%, respectively. The median unique molecular identifier (UMI) counts per cell for microglia cells in CX3CR1^GFP/GFP^ and C57BL/6J retinas were within the range of ~5000–10,000, and the median UMI counts per cell for whole retinal cells in two group were within the range of ~1000–2000. Sample demultiplexing, barcode processing and single-cell counting was performed using Cell Ranger (version 5.0.1). RNA reads were aligned with the mouse reference genome (refdata-gex-mm10-2020-A) using STAR alignment before calculating UMI counts. The raw output data were processed and analyzed using the Seurat package (version 5.0.1) in R software (version 4.3.1)^28^. The quality control was performed by filtering out cells identified with (1) a gene count less than 200, (2) a gene count greater than the maximum gene count × 90%, and (3) the top 15% of cells with the highest proportion of mitochondria reads. Cell cycle effects were corrected using the Seurat package, and potential doublets were removed using the DoubletFinder package (version 2.0.2)^29^. A total of 4575 and 1738 microglial cells from CX3CR1^GFP/GFP^ and C57BL/6J retinas were utilized for further analysis, respectively. For scRNA-seq analysis of whole retinal cells, 21,147 and 16,887 retinal cells from CX3CR1^GFP/GFP^ and C57BL/6J retinas were applied for subsequent analysis, respectively.

#### Data integration, dimensionality reduction and cell clustering

After normalizing the filtered gene-barcode matrices with the ‘NormalizedData’ function in R using the Seurat package, the top 2,000 highly variable genes were identified with the ‘FindVariableFeatures’ function. Furthermore, gene expression matrices were scaled and centered using the ‘ScaleData’ function followed by principal component (PC) analysis and uniform manifold approximation and projection (UMAP) dimension reduction using the top 20 PCs. To remove the batch effects across all samples, all data were processed using the ‘FindIntergrationAnchors’ and ‘IntegrateData’ functions in Seurat with default parameters. For microglial scRNA-seq dataset, clustering analysis was performed with the ‘FindNeighbors’ (first 20 PCs) and ‘FindClusters’ (resolution = 0.4) functions, which ultimately yielded 8 cell clusters. The cell annotation was conducted on the basis of the top marker gene expressions in each cluster, which were calculated using the ‘FindAllMarkers’ function.

Considering the low percentages of microglial cells in the retina (approximately 0.2% of total retinal cells^30^), we combined the microglial scRNA-seq dataset with the whole retinal cell scRNA-seq dataset to study the effects of microglia on other retinal cells, especially for cone photoreceptors and astrocytes. We randomly sampled 1,500 microglia from each microglial scRNA-seq dataset and integrated the data with the whole retinal cell scRNA-seq dataset after quality control. The data integration and dimensionality reduction analysis were performed as described above. Clustering analysis was conducted using the ‘FindNeighbors’ (first 20 PCs) and ‘FindClusters’ (resolution = 0.8) functions, and 22 cell clusters were identified. The cell annotation was performed on the basis of the DEG expression and well-known retinal cell markers from the literature^31–33^.

#### DEG identification, pathway enrichment analysis, GSEA and AUCell analysis

DEGs analysis was conducted using the ‘FindAllMarkers’ or ‘FindMarkers’ (min.pct = 0.1 and thresh.use = 0.25) functions with the Wilcoxon rank-sum test. DEGs were identified as |log_2_FC| >0.25 and adjusted *P* value <0.05 (refs. ^34,35^). The cluster-specific DEGs were used for KEGG pathway enrichment analysis using ShinyGO0.77 (http://bioinformatics.sdstate.edu/go/)^36^. The PPI among DEGs was analyzed using STRING (Version 12.0) and Cytoscape (version 3.10.1)^37^. To identify pathways that were induced or repressed in the cell clusters, we performed gene set enrichment analysis (GSEA) using gene sets from the KEGG database^38^. GSEA in specific cell clusters was conducted using the ‘GSEA’ and ‘gseaplot2’ functions from the GSEABase (Version 1.64.0), enrichplot (Version 1.22.0) and clusterProfiler (Version 4.10.0) packages in R software. Cell-level pathway activity scoring was performed using AUCell (Version 1.24.0) to calculate the activity level of pathways in each individual cell and visualize the activity scores on the UMAP. The gene set lists for the following pathways were obtained from the KEGG pathway database (https://www.genome.jp/kegg/kegg2.html): ‘TOLL_LIKE_RECEPTOR_SIGNALING_PATHWAY’ (mmu04620), ‘TNF_SIGNALING_PATHWAY’ (mmu04668), ‘PATHWAY_OF_NEURODEGENERATION’ (mmu05022) and ‘APOPTOSIS’ (mmu04210).

#### Analysis of cell–cell interactions

Cell–cell communications were performed with CellChat (Version 1.6.1)^39^. CellChat objects were generated from the Seurat object, and the data were preprocessed using the ‘subsetData’, ‘identifyOverExpressedGenes’ and ‘identifyOverExpressedInterations’ functions with default parameters. The ‘computeCommunProb’ function was then used to calculate communication probability and generate a communication network. The analysis was performed using the CellChatDB mouse database. To identify the cell–cell interaction among different microglia clusters, we utilized the ‘netVisual_chord_gene’ and ‘netVisual_individual’ functions. The identification of dominant senders, receivers, mediators and influencers in the intercellular communication network was calculated and visualized by the ‘netAnalysis_computeCentrality’ and ‘netAnalysis_signalingRole_network’ function, respectively.

To compare interaction strength in the different retinal cell clusters among CX3CR1^GFP/GFP^ and C57BL/6J retinas, we used the ‘compareInteractions’ function. To visualize the upregulated or downregulated signaling interactions originating from microglia clusters to astrocytes or photoreceptor clusters, we used the ‘netVisual_chord_gene’ and ‘netVisual_bubble’ functions. Furthermore, the ‘netAnalysis_signalingChanges_scatter’ function was used to visualize differential outgoing and incoming signaling in different cell clusters.

#### Pseudotime trajectory

Pseudotime trajectory analysis was performed by Monocle3 (Version 1.3.4)^40,41^. After constructing the cell data set (CDS) object, cells in microglia and cone clusters were selected for further analysis, respectively. Pseudotime information and trajectories were generated using the ‘learn_graph’ and ‘order_cells’ functions after performing dimensionality reduction and removal of batch effects. Root cells were selected on the basis of the real-time situation. Each cell was assigned a pseudotime value based on its projection on the UMAP graph obtained from the ‘learn_graph’ function. Finally, the results were visualized using the ‘plot_cells’ and ‘plot_genes_in_pseudotime’ functions in Monocle3.

#### TF module analysis

We used the SCENIC package (version 1.3.1) in R software to infer transcription factors (TFs) and gene regulatory networks for microglial clusters^42^. To achieve this, we constructed a gene expression matrix from 1000 cells (500 microglial cells randomly selected from each sample) based on raw counts. The RcisTarget databases ‘mm9-500bp-upstream-7species.mc9nr.feather’ and ‘mm9-tss-centered-10kb-7species.mc9nr.feather’, containing TF motif scores for gene promoters and around transcription start sites for the mouse reference genome, were used for reference. We used ‘runCorrelation’ and ‘GENIE3’ to calculate the correlation of candidate regulators. The downstream pipeline for constructing gene regulatory networks included ‘runSCENIC_1_coexNetwork2modules’, ‘runSCENIC_2_createRegulons’, ‘runSCENIC_3_scoreCells’ and ‘runSCENIC_4_aucell_binarize’. The projection of the area under the curve (AUC) and TF expression onto *t*-distributed stochastic neighbor embedding plots was visualized by ‘AUCell_plotTSNE’. Genotype-specific regulators were determined on the basis of the Regulon Specificity Score^42^.

### In vitro experiments

#### Cell lines

BV2 cells, a murine microglial cell line, were kindly provided by Prof. Haiwei Xu at Southwest Eye Hospital, Chongqing, China^43^. IMA2.1 cells, a murine astrocytes cell line, were purchased from Applied Biological Materials. 661W cells, a mouse cone photoreceptor-derived cell line, were generously provided by Dr. Muayyad Al-Ubaidi at the University of Oklahoma^44^. BV2, IMA2.1 and 661W cells were cultured in Dulbecco’s modified Eagle medium with 10% fetal bovine serum, 1% penicillin–streptomycin and GlutaMAX supplement in a 37 °C incubator under 5% CO_2_.

#### siRNA transfection

CX3CR1-, STAT3-, Ackr1-, Bmpr1a- and Bmpr1b-specific siRNAs were purchased from Invitrogen and Synbio Technologies. For siRNA transfection, BV2, IMA2.1 or 661W cells were seeded in a 12-well plate and cultured for 12 h, respectively. Afterwards, 10 µM siRNA was transfected with 9 µl Lipofectamine RNAiMAX Reagent (Life Technologies) for 6–8 h, followed by changing the culture medium before culturing cells for 36 h. Next, cells were collected for subsequent analysis. Meanwhile, cell supernatants from BV2 cells were also collected to stimulate IMA2.1 or 661W cells for 12 h, and then IMA2.1 or 661W cells were collected for further analysis. Gene-specific siRNAs used in this research are listed in Supplementary Table 1.

#### Colivelin TFA treatment and cytokine stimulation

IMA2.1 cells or 661W cells were seeded in a 12-well plate and cultured for 12 h before performing treatments. IMA2.1 cells were incubated with 100 ng/ml Colivelin TFA or PBS for 4 h, followed by collecting both cells and supernatants for further analysis. For the cytokine stimulation assay, IMA2.1 cells were treated with mouse TNF-α (25 ng/ml), CCL2 (100 ng/ml), CCL3 (100 ng/ml) and CCL4 (100 ng/ml) recombinant proteins for 16 h before collecting cells and supernatants for further analysis. 661W cells were treated with 25 ng/ml TNF-α, 100 ng/ml CCL2, 100 ng/ml CCL3, 100 ng/ml CCL4 or 100 ng/ml CXCL12 recombinant proteins for 12 h individually, and then cells were collected for apoptosis detection. To explore the effects of CCL/CXCL–ACKR1 signaling on cone photoreceptor apoptosis, 661W cells were transfected with siAckr1 or siCTR for 36 h before treating 100 ng/ml CCL2, 100 ng/ml CCL3, 100 ng/ml CCL4 or 100 ng/ml CXCL12 recombinant proteins or coculturing with cell inserts containing MACS-sorted microglia from CX3CR1^GFP/GFP^ or C57BL/6J for 12 h, and then 661W cells were collected for flow cytometry and qPCR analysis. To explore the roles of *Cxcl1*-dominant microglia on astrocytes, BV2 cells were seeded in the cell culture inserts in a 12-well plate for 12 h before treating with mouse CXCL1 (100 ng/ml) recombinant proteins for another 12 h. Then, the cell inserts containing BV2 cells were transferred into a 12-well plate with IMA2.1 cells that were previously transfected with siBmpr1a, siBmpr1b or siCTR for 36 h, followed by coculturing IMA2.1 cells with BV2 cells inserts for another 12 h before collecting IMA2.1 cells and supernatants for qPCR and ELISA analysis, respectively.

#### Cell apoptosis analysis

After different treatments, both 661W cells and the culture medium were collected into a new sterile tube and centrifuged at 1500 rpm for 5 min. Next, cells were rinsed with PBS one time before resuspending in 1× binding buffer. Meanwhile, FITC-conjugated Annexin V and propidium iodide were added to each sample according to the manual of apoptosis detection kit (Invitrogen, Abcam, R&D Systems or MedChemExpress), and cells were incubated for 5 min (for apoptosis kit from Abcam) or 15 min (for apoptosis kit from Invitrogen, R&D Systems or MedChemExpress) in the dark before detection using a BD FACSVia Flow Cytometer or BD FACSAria III Cell Sorter (BD Bioscience). All data were analyzed with BD FACSVia Research Software or FowJo V10 software.

#### Cytokine array assay

After siRNA transfection on BV2 cells or Colivelin TFA treatment on IMA2.1 cells, the supernatants were collected. Cytokine array assay was performed according to the user guide of the Proteome Profiler Mouse Cytokine Array Kit (R&D Systems). In brief, the membrane was blocked with Array Buffer 6 in a 4-well multi-dish for 1 h on a rocking platform shaker. Meanwhile, supernatant samples were prepared by adding 0.5 ml Array Buffer 4 and 15 μl of reconstituted Mouse Cytokine Array Panel A Detection Antibody Cocktail to 1 ml of supernatant, followed by incubation for 1 h at room temperature. Afterwards, the membrane was incubated with prepared supernatant sample overnight at 4 °C on a rocking platform shaker. After rinsing the membrane with 1× wash buffer three times, the membrane was incubated with 1× streptavidin-HRP in Array Buffer 6 for 30 min at room temperature on a rocking platform shaker. The membrane was incubated with 1 ml of Chemi Reagent Mix for 1 min before imaging with the ChemiDoc Imaging System (Bio-Rad). The integrated pixel density of each positive signal was measured using ImageJ software.

#### Immunoblotting

After different treatments, cells were collected, and total proteins were extracted with RIPA buffer (Abcam) containing a cocktail mixture of proteinase and phosphatase inhibitor cocktails (Roche). Western blotting was performed as described above. Primary antibodies, including rabbit CX3CR1 (Invitrogen, 1:500), rabbit p-STAT3 (Cell Signaling Technology, 1:1,000), rabbit STAT3 (Cell Signaling Technology, 1:1,000), mouse GAPDH (Millipore, 1:2,000) and mouse β-actin (Invitrogen, 1:2,000), were incubated overnight at 4 °C on a shaker. Goat anti-rabbit IgG and goat anti-mouse IgG (Invitrogen, 1:1,000) conjugated to horseradish peroxidase were applied as the secondary antibodies.

#### Quantitative real-time PCR

Cells were collected after different treatments, and the total RNA was extracted with TRIzol reagent (Invitrogen), and cDNA synthesis and qPCR were performed as described above. Gene-specific primers used for qPCR are listed in Supplementary Table 1.

#### Immunocytochemistry and confocal imaging

After the treatments, cells were fixed with 4% paraformaldehyde for 15 min, rinsed three times with PBS and then incubated with blocking buffer containing 3% normal donkey serum, 1% BSA and 0.3% Triton X-100 in PBS for 1 h, followed by incubation with rabbit p-STAT3 antibody (Cell Signaling Technology, 1:500), rat CD68 (Bio-Rad, 1:500), rat TNF-α (Santa Cruze, 1:200) and rabbit CXCL1 (Thermo Fisher Scientific, 1:200) with rabbit or goat Iba-1 (Wako, 1:500) or chicken GFAP (Abcam, 1:500) individually overnight at 4 °C. Afterwards, cells were rinsed with PBS three times and incubated with donkey anti-rabbit Alexa Flour 594 (Invitrogen, 1:500), donkey anti-rat Alexa Fluor 555 (Abcam, 1:500), donkey anti-rabbit Alexa Fluor 488 (Invitrogen, 1:500) and donkey anti-goat Alexa Fluor 488 (Invitrogen, 1:500) for 2 h before mounting slides with Dako fluorescence mounting medium. Images were captured by a Zeiss LSM 800 Upright Confocal Microscope (Zeiss). *Z*-stack images were acquired at 0.5-µm intervals using a 20× objective. An orthogonal projection was performed to generate the final image.

### Statistical analysis

All experiments involving imaging and quantification were repeated at least three times with similar results. Animal numbers used in each group are indicated in the figure legends. Data plotting and statistical tests were performed using GraphPad Prism software version 8.0. Data are represented as the mean ± s.e.m. and analyzed with unpaired two-tailed Student’s *t*-test or one-way analysis of variance (ANOVA) followed by Tukey’s multiple-comparisons test. In all graphs, statistical significance is described as *P < 0.05, **P < 0.01, ***P < 0.001, ****P < 0.0001.

## Results

### Microglial CX3CR1 deficiency induces glial reactivity and the selective vulnerability of cone photoreceptors in the retina

**Fig. 1:**
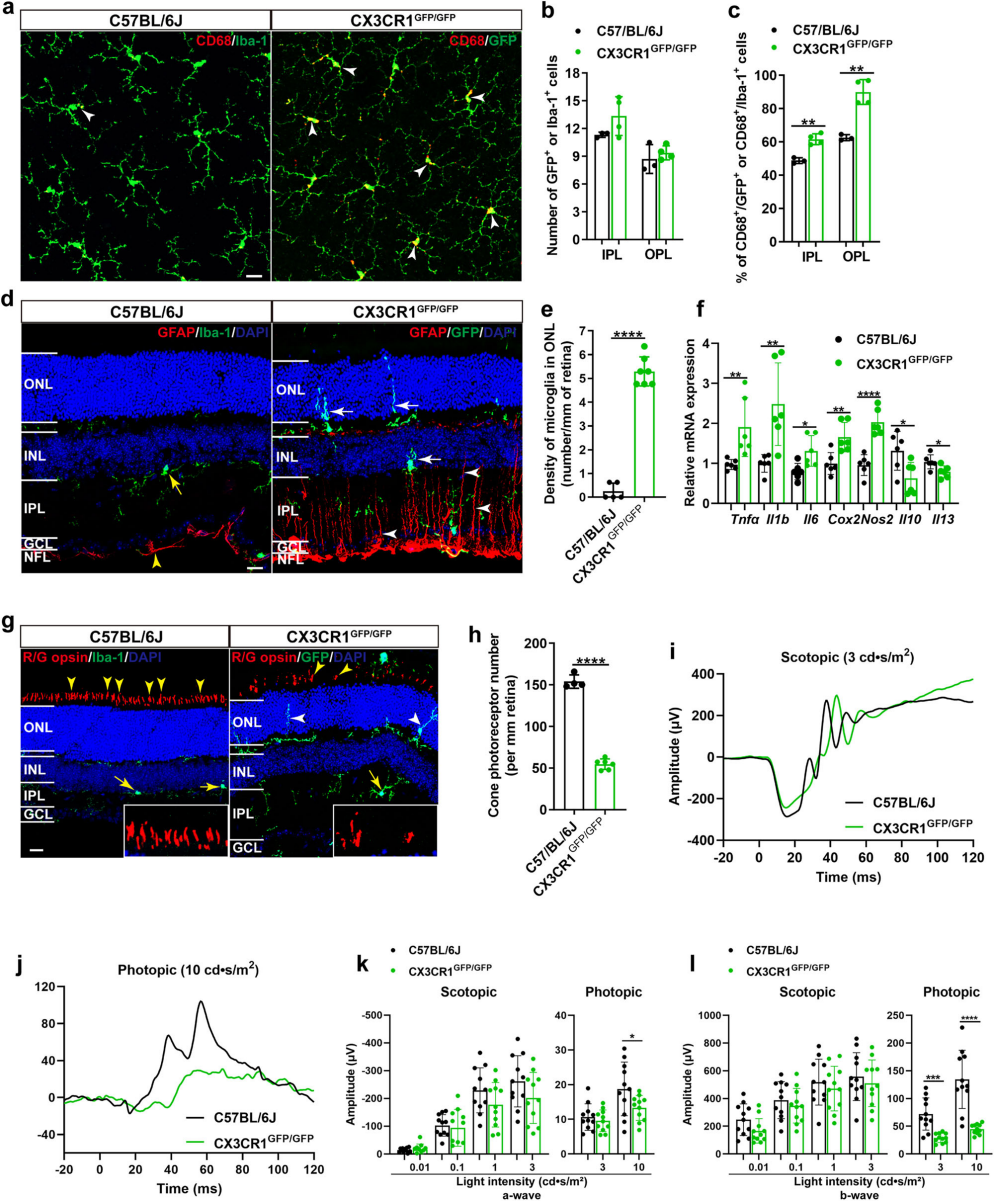
Microglial CX3CR1 deficiency induces glial reactivity and the selective vulnerability of cone photoreceptors in the retina. **a**, Retinal whole mounts from 6-week-old CX3CR1^GFP/GFP^ and C57BL/6J mice were stained with anti-CD68 and anti-Iba-1 antibodies. Representative confocal images focused on the OPL are shown. The white arrowheads indicate CD68^+^/GFP^+^ or CD68^+^/Iba-1^+^ microglia. **b**, **c**, Quantification of GFP^+^ or Iba-1^+^ (**b**) and CD68^+^/GFP^+^ or CD68^+^/Iba-1^+^ microglia (**c**) (*n* = 4 mice per group). **d** Retinal sections from CX3CR1^GFP/GFP^ and C57BL/6J mice were stained with anti-GFAP and anti-Iba-1 antibodies. The yellow arrow indicates a resting microglial cell, and the white arrows indicate the dendritic migration of activated microglia into the ONL and INL. The yellow arrowhead indicates a resting astrocyte, and the white arrowheads indicate the extension of the dendritic processes of activated astrocytes into the IPL. **e**, Quantification of activated microglia in the ONL of retinas from CX3CR1^GFP/GFP^ and C57BL/6J mice (*n* = 5–7 mice per group). **f**, qPCR analysis of proinflammatory molecules, including *Tnfα*, *Il1b*, *Il6*, *Cox2* and *Nos2*, and anti-inflammatory cytokines, including *Il10* and *Il13*, in CX3CR1^GFP/GFP^ and C57BL/6J mouse retinas (*n* = 6 mice per group). **g**, Retinal sections from CX3CR1^GFP/GFP^ and C57BL/6J mice were stained with anti-R/G opsin and anti-Iba-1 antibodies. Yellow arrowheads indicate cone photoreceptors. The boxed regions are highly magnified at the bottom, showing cone photoreceptors. Yellow arrows indicate resting microglia, and white arrowheads indicate the dendritic migration of activated microglia into the ONL. **h**, Quantification of cone photoreceptor cells in retinal sections (*n* = 4–6 mice per group). **i**, **j**, Representative ERG images of 6-week-old CX3CR1^GFP/GFP^ and C57BL/6J mice at 3 cd·s/m^2^ under scotopic conditions (**i**) and at 10 cd·s/m^2^ under photopic conditions (**j**). **k**, **l**, Amplitudes of ERG recordings under both scotopic and photopic conditions in 6-week-old CX3CR1^GFP/GFP^ and C57BL/6J mice (*n* = 11 mice per group). The data are presented as the mean ± s.e.m. and were analyzed via unpaired two-tailed Student’s *t*-tests (CX3CR1^GFP/GFP^ versus C57BL/6J, *P < 0.05, **P < 0.01, ***P < 0.001, ****P < 0.0001). INL, inner nuclear layer. Scale bar, 20 µm.

To explore whether microglial CX3CR1 deficiency caused any changes in the cellular morphology and function of the retina, we performed immunohistochemistry, qPCR and ERG on 6-week-old CX3CR1-deficient (CX3CR1^GFP/GFP^) mice; in these mice, the *Cx3cr1* gene was replaced with cDNA encoding green fluorescent protein. We observed that, under normal physiological conditions, microglia were distributed in the outer plexiform layer (OPL), inner plexiform layer (IPL) and ganglion cell layer (GCL) of the retina and did not express CD68, which is a marker of activated microglia. However, under conditions of microglial CX3CR1 deficiency, the number of activated microglia (CD68^+^) in both the OPL and IPL was greater than that in these regions of age-matched C57BL/6J mouse retinas (Fig. 1a–c). Moreover, the dendritic processes of activated microglia in CX3CR1-deficient retinas extended from the normal stratum within the OPL into the outer nuclear layer (ONL) (Fig. 1d, e). Interestingly, we observed sustained microglial activation over a long period of time (up to P360) in CX3CR1^GFP/GFP^ retinas compared with age-matched C57BL/6J controls at six different stages (Supplementary Fig. 1a, b), suggesting that CX3CR1 deficiency triggers chronic disruption of microglial homeostatic regulation rather than transient activation.

Moreover, microglial CX3CR1 knockout significantly increased proinflammatory molecules (*Tnfα*, *Il1b*, *Il6*, *Cox2* and *Nos2*) expression but decreased anti-inflammatory cytokine (*Il10* and *Il13*) expression in CX3CR1-deficient retinas compared with C57BL/6J mouse retinas (Fig. 1f).

Furthermore, we noted that GFAP immunoreactivity, which is a marker of astrocyte activation, was increased in CX3CR1-deficient retinas (Fig. 1d) and that these activated astrocytes exhibited hypertrophic cell bodies and processes that migrated to the IPL from their normal location within the nerve fiber layer (NFL) (Fig. 1d). To quantify proinflammatory (A1) and anti-inflammatory (A2) reactive astrocytes, we performed qPCR to measure the expression of A1-specific genes (*C3*, *H2-T23*, *Ggta1*, *Fbln5*, *Fkbp5* and *Gbp2*), which are conventionally associated with the production of proinflammatory factors and neurotoxins^45,46^, and A2-specific genes (*Clcf1*, *Cd109*, *Ptgs2* and *Cd14*), which are involved in the production of anti-inflammatory cytokines and neurotrophic factors^45,46^. We observed that A1-specific genes were upregulated in CX3CR1-deficient retinas, whereas A2-specific genes were downregulated (Supplementary Fig. 1c).

In addition, we observed that most cone photoreceptors had markedly shorter outer segments that were recognized by an anti-R/G opsin antibody and that the number of cones was significantly lower in CX3CR1-deficient retinas than in C57BL/6J retinas (Fig. 1g, h); these results indicated cone photoreceptor degeneration. Functionally, we observed significantly reduced amplitudes and delayed latencies of photopic a- and b-waves of ERG in CX3CR1-deficient mice (Fig. 1i–l and Supplementary Fig. 1d, e), indicating compromised cone function. However, we did not detect any significant change in the dark-adapted a- or b-wave amplitudes or latency (Fig. 1i–l and Supplementary Fig. 1d, e), suggesting that rod function was relatively stable.

Together, these findings indicate that microglial CX3CR1 deficiency activates both microglia and astrocytes and induces the selective vulnerability of cone photoreceptors in the retina.

### STAT3 is a potential contributor to microglial neurotoxicity in CX3CR1-deficient retinas

**Fig. 2:**
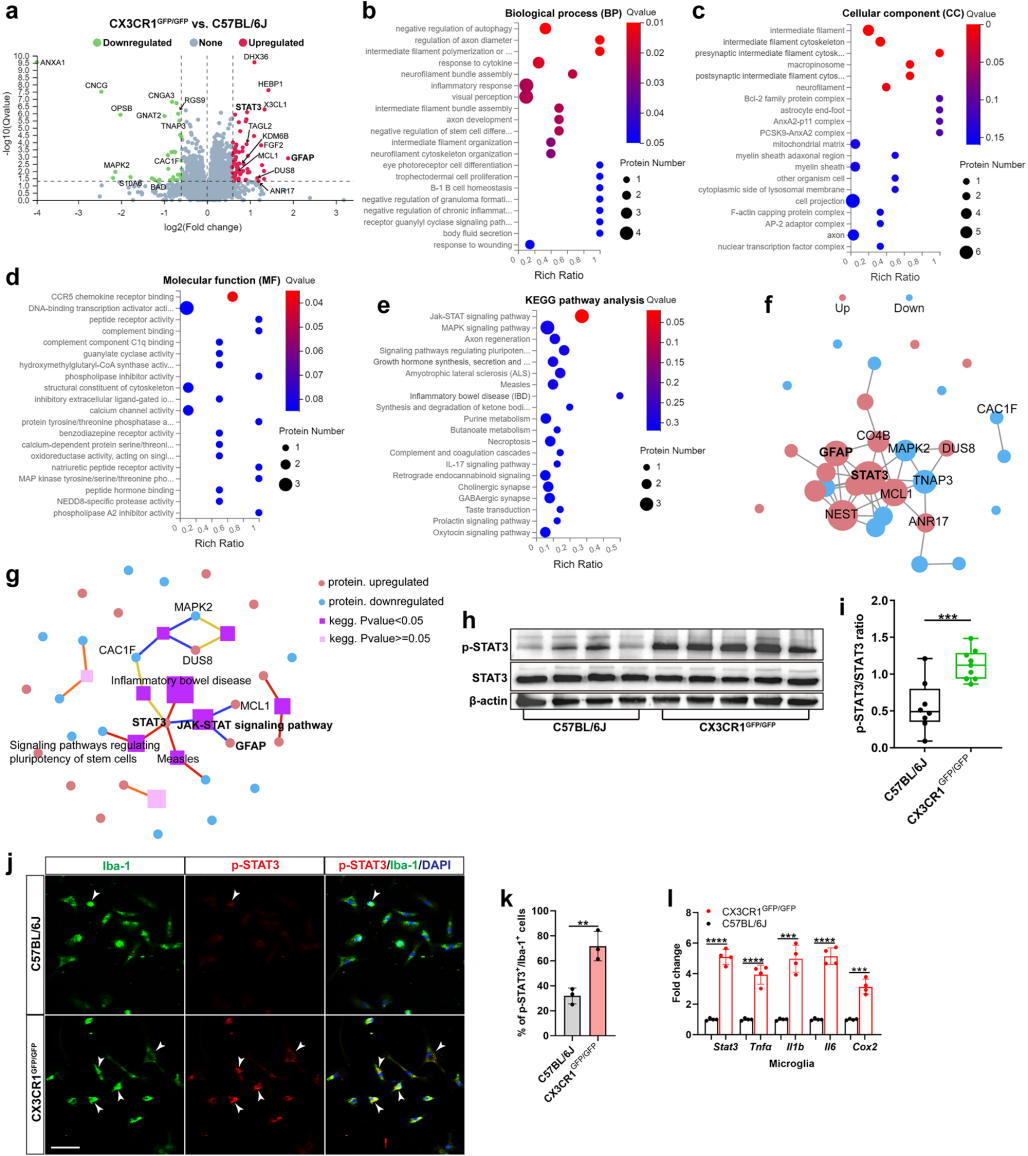
STAT3 contributes to microglial neurotoxicity in CX3CR1-deficient retinas. **a**, Volcano plot showing DEPs in retinas from 6-week-old CX3CR1^GFP/GFP^ and C57BL/6J mice (*n* = 5 mice per group). Red dots indicate upregulated proteins (FC >1.5), and green dots indicate downregulated proteins (FC <1/1.5), with a *Q* value <0.05. **b**–**d**, GO analysis of DEPs, shown separately for biological process (**b**), cellular component (**c**) and molecular function (**d**), between CX3CR1^GFP/GFP^ retinas and C57BL/6J retinas. **e**, KEGG pathway analysis of DEPs between CX3CR1^GFP/GFP^ retinas and C57BL/6J retinas. **f**, PPI analysis of DEPs. Pink dots indicate upregulated proteins, and blue dots represent downregulated proteins. **g**, KEGG network analysis of DEPs. The purple squares indicate the KEGG pathways, and a deeper color indicates greater significance. The red dots indicate upregulated DEPs, and the blue dots indicate downregulated DEPs. **h**, **i**, Western blotting analysis (**h**) and quantification of p-STAT3/STAT3 expression (**i**) in retinas from 6-week-old CX3CR1^GFP/GFP^ and C57BL/6J mice (*n* = 8–9 mice per group). **j**, Colocalization of p-STAT3 and Iba-1 in microglia from CX3CR1^GFP/GFP^ and C57BL/6J retinas at 6 weeks of age. The white arrowheads indicate p-STAT3^+^/Iba-1^+^ microglia. Scale bar, 50 µm. **k**, Quantification of the percentages of p-STAT3^+^/Iba-1^+^ microglia in **j**. **l**, qPCR analysis of *Stat3* and proinflammatory molecule expression in microglia from CX3CR1^GFP/GFP^ and C57BL/6J retinas at 6 weeks of age. The results shown represent three to four independent experiments. The data are presented as the mean ± s.e.m. and were analyzed via unpaired two-tailed Student’s *t*-test (**P < 0.01, ***P < 0.001, ****P < 0.0001).

To investigate the mechanisms underlying the observed selective cone cell vulnerability, we performed liquid chromatography‒tandem mass spectrometry to identify DEPs between CX3CR1-deficient and C57BL/6J retinas. In total, 95 DEPs, including 63 upregulated and 32 downregulated DEPs, were identified (Fig. 2a). Inflammation-related genes, including STAT3, GFAP, MCL1 and KDM6B, were significantly upregulated in CX3CR1-deficient retinas (Fig. 2a). GO analysis of the DEPs revealed that most of the DEPs were associated with cell proliferation, differentiation, inflammation, autophagy and apoptosis processes (Fig. 2b–d). KEGG pathway analysis of the DEPs revealed significant enrichment of the JAK–STAT signaling pathway (*Q* value: 0.026) (Fig. 2e), which is involved in mediating cell survival, differentiation, apoptosis and inflammation^47,48^. PPI network analysis of the DEPs revealed that STAT3 is a critical molecule that tightly interacts with other DEPs (Fig. 2f), such as GFAP. Consistent with the PPI analysis results, KEGG network analysis of the DEPs also revealed that STAT3 interacted strongly with other DEPs and with signaling pathways, particularly the JAK–STAT signaling pathway (Fig. 2g). Furthermore, western blotting analysis confirmed that the level of phosphorylated STAT3 (p-STAT3) was significantly greater in CX3CR1-deficient retinas than in C57BL/6J control retinas (Fig. 2h, i). Specifically, we observed increased expression of p-STAT3 (Fig. 2j, k) and proinflammatory molecules in primary microglia that were sorted from CX3CR1-deficient retinas (Fig. 2l). These data suggest that STAT3 could be a potential downstream molecule of CX3CR1 that modulates inflammation and selective cone cell death.

### Gain or loss of function of STAT3 in regulating glial reactivity and the selective vulnerability of cone photoreceptors in CX3CR1-deficient retinas

**Fig. 3:**
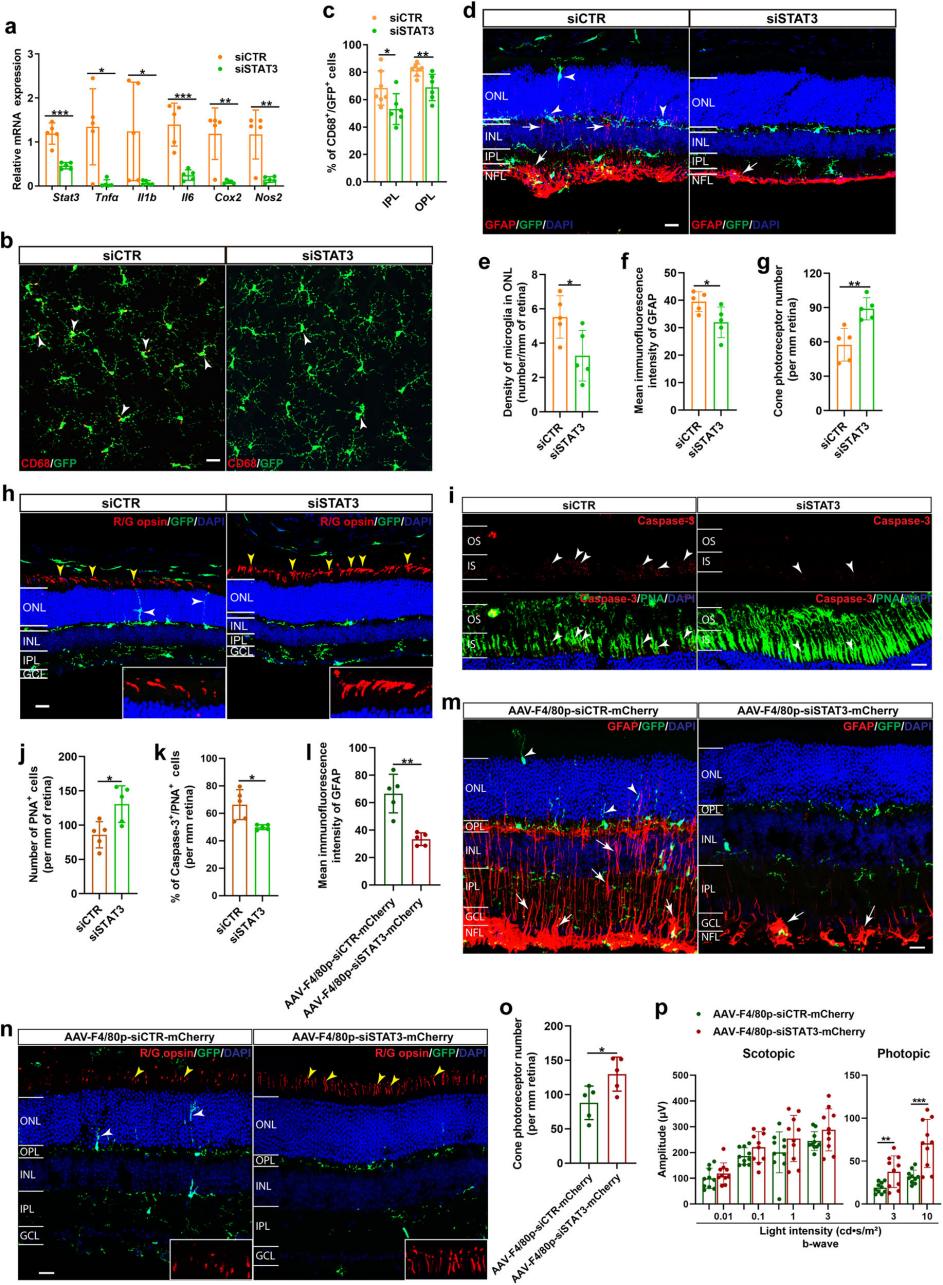
STAT3 knockdown ameliorates microglial neurotoxicity induced by CX3CR1 deficiency. **a**, qPCR analysis of *Stat3* and proinflammatory molecule expression in retinas from 6-week-old CX3CR1^GFP/GFP^ mice treated with siSTAT3 or siCTR (*n* = 5 mice per group). **b**, Retinal whole mounts from CX3CR1^GFP/GFP^ mice treated with siSTAT3 or siCTR were stained with an anti-CD68 antibody. The white arrowheads indicate CD68-positive microglia. **c**, Quantification of CD68^+^/GFP^+^ microglia (*n* = 6–7 mice per group). **d**, Retinal sections from siSTAT3- or siCTR-treated CX3CR1^GFP/GFP^ mice were stained with an anti-GFAP antibody. The white arrows indicate the extension of the dendritic processes of activated astrocytes into the IPL, and the white arrowheads indicate the dendritic migration of activated microglia into the ONL. **e**, Quantification of microglial cell density in the ONL of retinas from CX3CR1^GFP/GFP^ mice treated with siSTAT3 or siCTR (*n* = 5 mice per group). **f**, Quantification of the mean immunofluorescence intensity of GFAP in retinal sections (*n* = 5 mice per group). **g**, Quantification of cone photoreceptors in retinas from siSTAT3- or siCTR-treated CX3CR1^GFP/GFP^ mice (*n* = 5 mice per group). **h**, Retinal sections from siSTAT3- or siCTR-treated CX3CR1^GFP/GFP^ mice were stained with an anti-R/G opsin antibody. Yellow arrowheads indicate cone photoreceptors. The boxed regions are shown at higher magnification at the bottom. The white arrowheads show the dendritic migration of activated microglia into the ONL. **i**, Retinal sections from CX3CR1^GFP/GFP^ mice after treatment with siSTAT3 or siCTR were stained with anti-Caspase-3 and anti-PNA antibodies. The white arrowheads indicate Caspase-3^+^/PNA^+^ cells. **j**, **k**, Quantification of the number of PNA^+^ cells (**j**) and percentages of Caspase-3^+^/PNA^+^ cells (**k**) in retinal sections (*n* = 5 mice per group). **l**, Quantification of the mean immunofluorescence intensity of GFAP (*n* = 5 mice per group). **m**, Retinal sections from CX3CR1^GFP/GFP^ mice treated with AAV-F4/80p-siSTAT3-mCherry or AAV-F4/80p-siCTR-mCherry were stained with an anti-GFAP antibody. The white arrowheads show the migration of activated microglia in the ONL, and the white arrows indicate the dendritic extension of activated astrocytes into the IPL from the NFL. **n**, Retinal sections from 6-week-old CX3CR1^GFP/GFP^ mice treated with AAV-F4/80p-siSTAT3-mCherry or AAV-F4/80p-siCTR-mCherry were stained with an anti-R/G opsin antibody. The white arrowheads indicate the migration of activated microglia in the ONL, and the yellow arrowheads indicate the R/G opsin^+^ cone photoreceptors. **o**, Quantification of the number of R/G opsin^+^ cone photoreceptors in **n** (*n* = 5 mice per group). **p**, ERG recordings of CX3CR1^GFP/GFP^ mice at 6 weeks of age treated with AAV-F4/80p-siSTAT3-mCherry or AAV-F4/80p-siCTR-mCherry (*n* = 10 mice per group). Scale bar, 20 µm. The data are presented as the mean ± s.e.m. and were analyzed via unpaired two-tailed Student’s *t*-tests (*P < 0.05, **P < 0.01, ***P < 0.001).

To confirm the role of STAT3 in selective cone cell loss, we next genetically knocked down STAT3 expression in CX3CR1-deficient retinas by using a STAT3-specific siRNA (Fig. 3a and Supplementary Fig. 2a–c). We found that, compared with the siCTR control, STAT3 knockdown significantly decreased proinflammatory molecule expression (Fig. 3a), suggesting that inflammation was inhibited. Morphologically, we observed that the number of activated microglia (CD68^+^) in the ONL, OPL and IPL of CX3CR1-deficient retinas was significantly lower (Fig. 3b–e) and that fewer dendritic processes of activated microglia extended into the ONL from the OPL in CX3CR1-deficient retinas following STAT3 knockdown (Fig. 3d, e) than in those of siCTR-treated control retinas, indicating that microglial activation was suppressed.

Moreover, we observed that STAT3 knockdown markedly decreased GFAP immunoreactivity; inhibited the extension of astrocyte processes into the IPL from the NFL (Fig. 3d, f); and elevated the expression of A2-specific signature genes, including *Cd109*, *Ptgs2* and *S100a10* (Supplementary Fig. 2d), in CX3CR1-deficient retinas. These results indicated that astrocyte reactivity was suppressed. Furthermore, we observed that STAT3 knockdown notably increased the number of cone photoreceptors, as revealed by an antibody against R/G opsin or PNA (Fig. 3g–j), but decreased the percentage of apoptotic cone photoreceptors (Caspase-3^+^/PNA^+^) in CX3CR1-deficient retinas (Fig. 3i, k). These results indicated the increased survival of cones.

To directly verify the role of STAT3 in microglia, we knocked down STAT3 specifically in microglia via the intravitreal injection of AAV9-packaged siSTAT3 under the control of the microglia-specific promoter F4/80p^17,18^ into CX3CR1-deficient mice (Supplementary Fig. 2e, f). We observed that microglial STAT3 knockdown significantly inhibited astrocyte reactivity (Fig. 3l, m) and increased cone cell survival (Fig. 3n, o) and function, as monitored by photopic ERGs (Fig. 3p and Supplementary Fig. 2g). Together, these results further support the hypothesis that microglial STAT3 directly regulates astrocyte reactivity and cone survival in CX3CR1-deficient retinas.

To further confirm the role of STAT3, we increased STAT3 activity in CX3CR1-deficient retinas via the intraperitoneal injection of Colivelin TFA, which is a selective activator of STAT3 (Supplementary Fig. 3a–c). Compared with PBS treatment, Colivelin TFA treatment significantly increased proinflammatory molecule expression (Supplementary Fig. 3d) and enhanced microglial activation, as indicated by increased numbers of microglia, increased numbers of activated microglia (CD68^+^) (Supplementary Fig. 3e–g) and increased numbers of dendritic processes of activated microglia migrating into the ONL from the OPL in Colivelin TFA-treated CX3CR1-deficient retinas compared with PBS-treated retinas (Supplementary Fig. 3h, i). Moreover, compared with the control conditions, Colivelin TFA treatment further increased astrocyte reactivity, as indicated by increased GFAP immunoreactivity, increased numbers of astrocytes with hypertrophic somas and thickened dendritic processes (Supplementary Fig. 3h, j), and significantly increased expression of A1 signature genes, including *C3*, *Iigp1*, *Fkbp5* and *Gbp2*, in CX3CR1-deficient retinas (Supplementary Fig. 3k). Finally, we observed that, compared with PBS treatment, Colivelin TFA treatment notably decreased the number of cone photoreceptors in CX3CR1-deficient retinas (Supplementary Fig. 3l, m), suggesting that Colivelin TFA-induced increases in STAT3 activity exacerbate proinflammatory responses and cause additional cone photoreceptor loss in the CX3CR1-deficient retina. To further establish the causal role of STAT3 in microglial neurotoxicity following CX3CR1 deficiency, we administered siSTAT3 under the control of the microglia-specific promoter F4/80p through intravitreal injection to achieve knockdown of microglial STAT3 signal, followed by systemic STAT3 activation via intraperitoneal injection of Colivelin TFA, a selective STAT3 activator. We observed that STAT3 knockdown significantly attenuated CX3CR1 deficiency-induced microglial activation and cone photoreceptor loss (Supplementary Fig. 3n–r). Notably, pharmacological reactivation of STAT3 largely reversed these protective effects, leading to microglial activation and cone cell death (Supplementary Fig. 3n–r), further confirming the critical role of STAT3 in microglial neurotoxicity.

### Microglial STAT3 regulates astrocyte reactivity and cone photoreceptor apoptosis

**Fig. 4:**
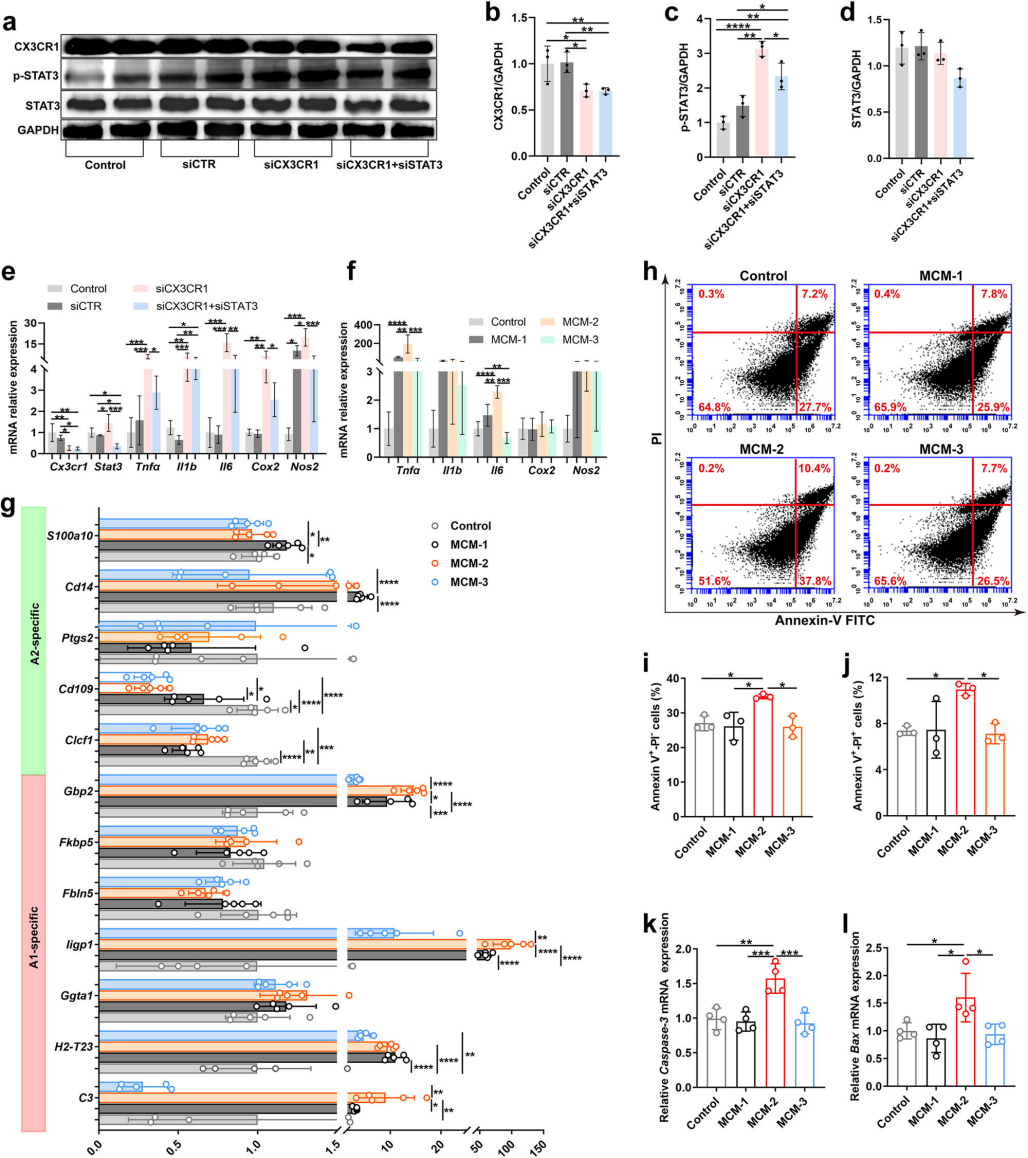
Microglial STAT3 regulates astrocyte activation and cone photoreceptor loss. **a**–**d**, Western blotting analysis (**a**) and quantification of CX3CR1 (**b**), p-STAT3 (**c**) and STAT3 expression (**d**) in BV2 cells transfected with siSTAT3 and siCX3CR1 or siCX3CR1 and siCTR. **e**, qPCR analysis of the expression of the proinflammatory molecules *Cx3cr1* and *Stat3* in BV2 cells after transfection with siSTAT3 and siCX3CR1 or siCX3CR1 and siCTR. **f**, qPCR analysis of proinflammatory cytokines in IMA2.1 cells treated with MCM-3, MCM-2 or MCM-1. **g**, qPCR analysis of A1- and A2-specific gene expression in IMA2.1 cells treated with MCM-3, MCM-2 or MCM-1. **h**–**j**, Flow cytometry analysis (**h**) and quantification of the percentages of Annexin-V^+^/PI^−^ (**i**) and Annexin-V^+^/PI^+^ (**j**) cells among 661W cells after treatment with MCM-3, MCM-2 or MCM-1. **k**–**l**, qPCR analysis of *Caspase-3* (**k**) and *Bax* (**l**) expression in 661W cells treated with MCM-3, MCM-2 or MCM-1. The results shown represent three to five independent experiments. The data are presented as the mean ± s.e.m. and were analyzed via one-way ANOVA with Tukey’s multiple comparison test (*P < 0.05, **P < 0.01, ***P < 0.001, ****P < 0.0001).

To further confirm the role of microglial STAT3, we performed in vitro experiments. We found that CX3CR1 knockdown in BV2 murine microglia significantly increased the levels of p-STAT3 (Fig. 4a–d) and proinflammatory molecules (Fig. 4e), and these effects were reversed by STAT3 knockdown (Fig. 4a–e), confirming that STAT3 is a critical downstream effector of CX3CR1. To investigate whether microglial STAT3 regulates astrocyte reactivity, we cultured IMA2.1 murine astrocytes in conditioned medium collected from BV2 cells after transfection with siCTR (MCM-1), siCX3CR1 (MCM-2) or both siCX3CR1 and siSTAT3 (MCM-3). We found that MCM-2-treated IMA2.1 cells released higher levels of proinflammatory cytokines, including *Tnfα* and *Il6*, and A1-specific genes, including *C3*, *Iigp1* and *Gbp2*, than MCM-1-treated IMA2.1 cells did, and these effects were suppressed in MCM-3-treated IMA2.1 cells (Fig. 4f, g). These data confirmed that microglial STAT3 mediated astrocyte reactivity. To examine whether microglial STAT3 induced cone cell apoptosis, we cultured 661W cone photoreceptor cells in MCM-1, MCM-2 or MCM-3. We found that MCM-2 treatment significantly increased the number of Annexin-V^+^/PI^+^ and Annexin-V^+^/PI^-^ cells (Fig. 4h–j) and the expression of proapoptotic genes in 661W cells (Fig. 4k, l), and these effects were largely reversed in MCM-3-treated 661W cells. Taken together, these data indicate that microglial STAT3 directly regulates astrocyte reactivity and cone cell apoptosis.

### Astrocytic STAT3 regulates astrocyte reactivity and cone photoreceptor apoptosis

**Fig. 5:**
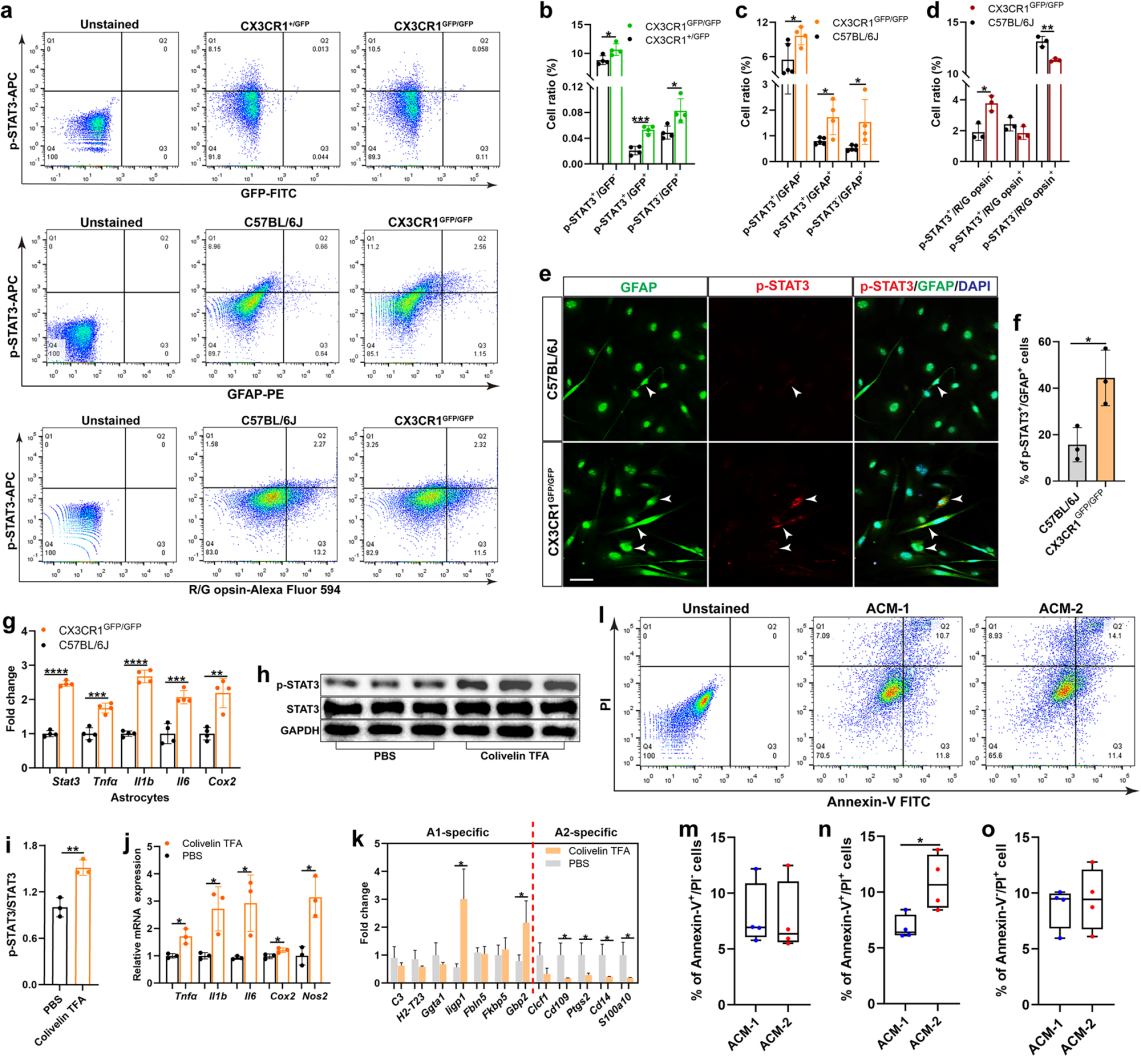
Astrocytic STAT3 regulates cone photoreceptor loss. **a**, Flow cytometry analysis of p-STAT3 expression in microglia (CD11b^+^), astrocytes (GFAP^+^) and cone photoreceptors (R/G opsin^+^) from the retinas of CX3CR1^GFP/GFP^ and CX3CR1^+/GFP^ or C57BL/6J mice at 6 weeks of age (*n* = 3–4 mice per group). **b**, Quantification of the percentages of p-STAT3^+^/GFP^−^, p-STAT3^+^/GFP^+^ and p-STAT3^−^/GFP^+^ cells in **a**. **c**, Quantification of the percentages of p-STAT3^+^/GFAP^−^, p-STAT3^+^/GFAP^+^ and p-STAT3^−^/GFAP^+^ cells in **a**. **d**, Quantification of the percentages of p-STAT3^+^/R/G-opsin^−^, p-STAT3^+^/R/G opsin^+^ and p-STAT3^−^/R/G opsin^+^ cells in **a**. **e**, Colocalization of p-STAT3 and GFAP in astrocytes from the retinas of 6-week-old CX3CR1^GFP/GFP^ or C57BL/6J mice. The white arrowheads indicate p-STAT3^+^/GFAP^+^ cells. Scale bar, 50 µm. **f**, Quantification of the percentages of p-STAT3^+^/GFAP^+^ cells in **e**. **g**, qPCR analysis of *Stat3* and proinflammatory molecule expression in astrocytes from the retinas of 6-week-old CX3CR1^GFP/GFP^ or C57BL/6J mice. **h**, **i**, Western blotting analysis (**h**) and quantification of p-STAT3/STAT3 expression levels (**i**) in IMA2.1 cells treated with Colivelin TFA or PBS. **j**, qPCR analysis of proinflammatory molecules in IMA2.1 cells after Colivelin TFA or PBS treatment. **k**, qPCR analysis of A1- and A2-specific gene expression in IMA2.1 cells treated with Colivelin TFA or PBS. **l**‒**o**, Flow cytometry analysis (**l**) and quantification of the percentages of Annexin-V^+^/PI^−^ (**m**), Annexin-V^+^/PI^+^ (**n**) and Annexin-V^−^/PI^+^ (**o**) cells among 661W cells treated with ACM-2 or ACM-1. The results shown represent three to four independent experiments. The data are presented as the mean ± s.e.m. and were analyzed via unpaired two-tailed Student’s *t*-tests (*P < 0.05, **P < 0.01, ***P < 0.001, ****P < 0.0001).

To evaluate whether microglial CX3CR1 deficiency upregulated STAT3 in astrocytes, we performed flow cytometry analysis of whole CX3CR1-deficient retinas (Fig. 5a–d). We found that the number of p-STAT3^+^ cells was significantly greater in the CX3CR1-deficient retinas than in the CX3CR1^+/GFP^ or C57BL/6J control retinas (Fig. 5a–d). In particular, the percentages of both p-STAT3^+^ microglia and p-STAT3^+^ astrocytes were markedly greater in the CX3CR1-deficient retinas than in the control retinas (Fig. 5a–c). However, we did not observe any significant change in the percentage of p-STAT3^+^ cone cells in CX3CR1-deficient retinas (Fig. 5a, d). Similarly, we observed that the expression levels of p-STAT3 and proinflammatory molecules in astrocytes from CX3CR1-deficient retinas were significantly increased (Fig. 5e–g). These results indicate that microglial CX3CR1 deficiency increased astrocytic STAT3 and astrocyte reactivity.

To confirm whether astrocytic STAT3 plays the same role as microglial STAT3 (Fig. 4), we activated STAT3 in IMA2.1 cells with Colivelin TFA (Fig. 5h, i). We found that Colivelin TFA treatment markedly increased the levels of proinflammatory molecules (Fig. 5j) and A1-specific signature genes in IMA2.1 cells but inhibited A2-specific gene expression (Fig. 5k), suggesting that astrocytic STAT3 is capable of mediating astrocyte reactivity.

To examine whether astrocytic STAT3 induced cone cell apoptosis, we cultured 661W cone cells in conditioned medium collected from Colivelin TFA-treated IMA2.1 cells (ACM-2) or PBS-treated IMA2.1 cells (ACM-1). Flow cytometry analysis revealed that ACM-2 treatment significantly increased the number of Annexin-V^+^/PI^+^ 661W cells compared with ACM-1 treatment (Fig. 5l–o). Collectively, these findings indicate that astrocytic STAT3 induces astrocyte reactivity and subsequently drives cone cell apoptosis.

### TNF-α, CCL2, CCL3, CCL4 and CXCL12 derived from activated microglia and reactive astrocytes are associated with selective cone cell apoptosis

**Fig. 6:**
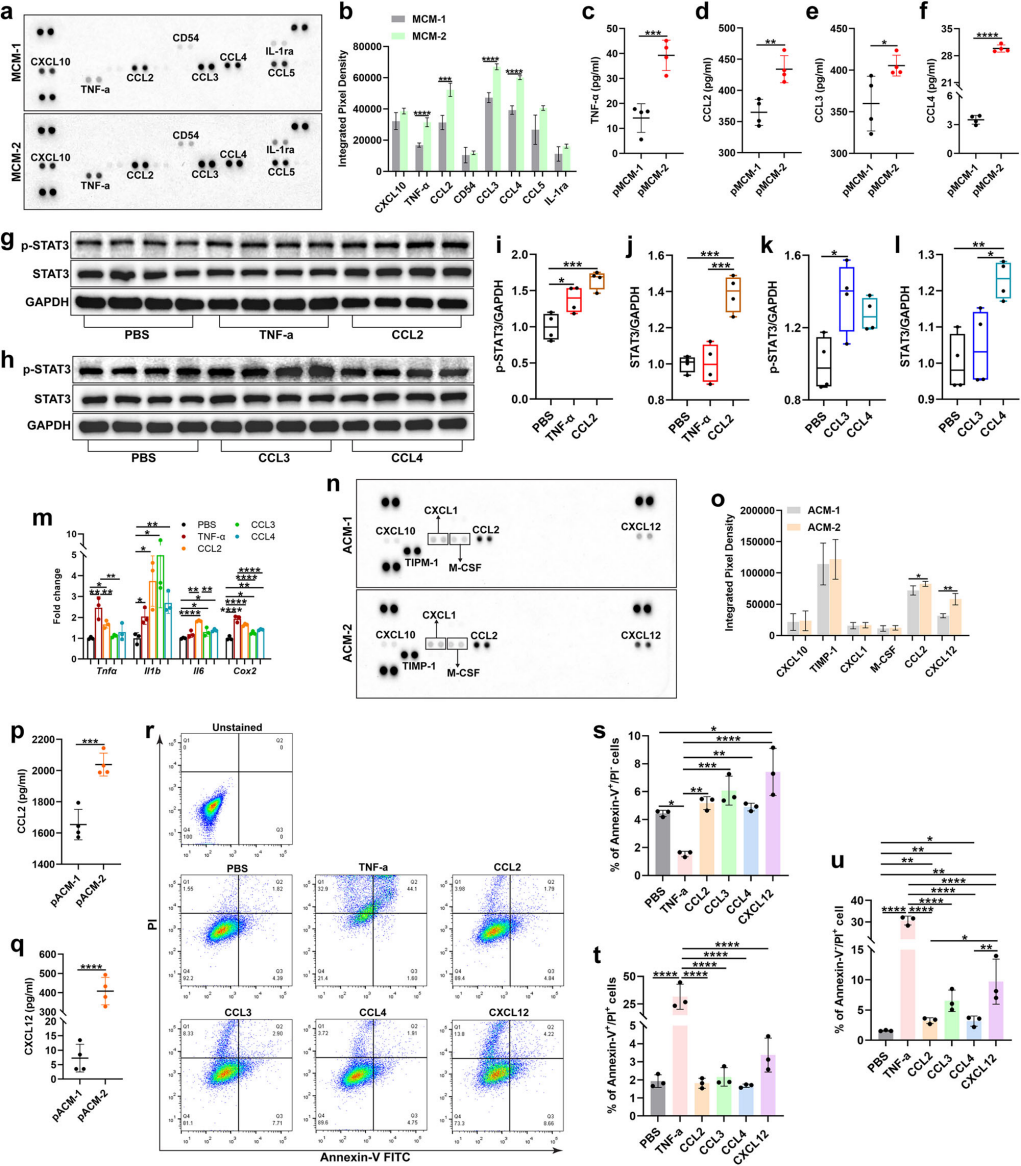
Proinflammatory cytokines derived from activated microglia and astrocytes are associated with cone cell apoptosis. **a**,**b**, Cytokine array analysis (**a**) and quantification of differentially expressed cytokines/chemokine levels (**b**) in MCM-2 or MCM-1. **c**–**f**, ELISA analysis of TNF-α (**c**), CCL2 (**d**), CCL3 (**e**) and CCL4 (**f**) levels in pMCM-2 or pMCM-1. **g**–**l**, Western blotting analysis (**g** and **h**) and quantification of p-STAT3 (**i** and **k**) and STAT3 (**j** and **l**) expression in IMA2.1 cells treated with recombinant TNF-α (25 ng/ml), CCL2 (100 ng/ml), CCL3 (100 ng/ml) or CCL4 (100 ng/ml) proteins. **m**, qPCR analysis of proinflammatory cytokine expression in IMA2.1 cells treated as described in **g**–**l**. **n**, **o**, Cytokine array analysis (**n**) and quantification of differentially expressed cytokines/chemokines (**o**) in ACM-2 or ACM-1. **p**, **q**, ELISA of CCL2 (**p**) and CXCL12 expression (**q**) in pACM-2 or pACM-1. **r**‒**u**, Flow cytometry analysis (**r**) and quantification of the percentages of Annexin-V^+^/PI^−^ (**s**), Annexin-V^+^/PI^+^ (**t**) and Annexin-V^−^/PI^+^ (**u**) cells in 661W cells treated with recombinant TNF-α (25 ng/ml), CCL2 (100 ng/ml), CCL3 (100 ng/ml), CCL4 (100 ng/ml) or CXCL12 (100 ng/ml) proteins. The results shown represent three to four independent experiments. The data are presented as the mean ± s.e.m. and were analyzed via one-way ANOVA with Tukey’s multiple comparison test or unpaired two-tailed Student’s *t*-test (*P < 0.05, **P < 0.01, ***P < 0.001, ****P < 0.0001).

In the subsections above, we demonstrated that toxic effects of activated microglia and reactive astrocytes triggered selective cone photoreceptor apoptosis both in vitro and in vivo. To identify such toxicity, we performed a cytokine array with MCM-2 or ACM-2 medium. The array results revealed that the levels of TNF-α, CCL2, CCL3 and CCL4 were significantly elevated in MCM-2 compared with MCM-1 (Fig. 6a, b). Consistently, we found that the protein levels of TNF-α, CCL2, CCL3 and CCL4 were markedly greater in culture media collected from primary microglia from CX3CR1-deficient retinas (pMCM-2) than in those from C57BL/6J retinas (pMCM-1) (Fig. 6c–f). To verify the roles of these individual cellular products, we treated IMA2.1 cells with recombinant TNF-α, CCL2, CCL3 or CCL4 protein. Compared with PBS treatment, all four recombinant protein-treated groups exhibited markedly increased levels of p-STAT3, STAT3 (Fig. 6g–l) and proinflammatory molecules (Fig. 6m).

Moreover, we found that the levels of the chemokines CCL2 and CXCL12 were markedly greater in ACM-2 medium than in ACM-1 medium (Fig. 6n, o). Similarly, we observed higher levels of CCL2 and CXCL12 in culture media collected from primary astrocytes from CX3CR1-deficient retinas (pACM-2) than from C57BL/6J retinas (pACM-1) (Fig. 6p, q).

Together, these results suggest that the release of TNF-α, CCL2, CCL3 and CCL4 from CX3CR1-deficient microglia directly triggers astrocyte reactivity, which subsequently leads to the release of the chemokines CCL2 and CXCL12.

Finally, to confirm the neurotoxicity of these identified factors, we treated 661W cone cells with recombinant TNF-α, CCL2, CCL3, CCL4 or CXCL12 protein. Annexin-V/PI apoptosis analysis revealed that all five recombinant proteins significantly induced greater 661W cell apoptosis than did the PBS-treated controls (Fig. 6r–u). These results suggest that the cytokines and chemokines that are released by activated microglia and reactive astrocytes are capable of inducing cone cell apoptosis.

### *Tnf*-dominant microglia contribute to increased microglial STAT3 levels and neurotoxicity in CX3CR1-deficient retinas

**Fig. 7:**
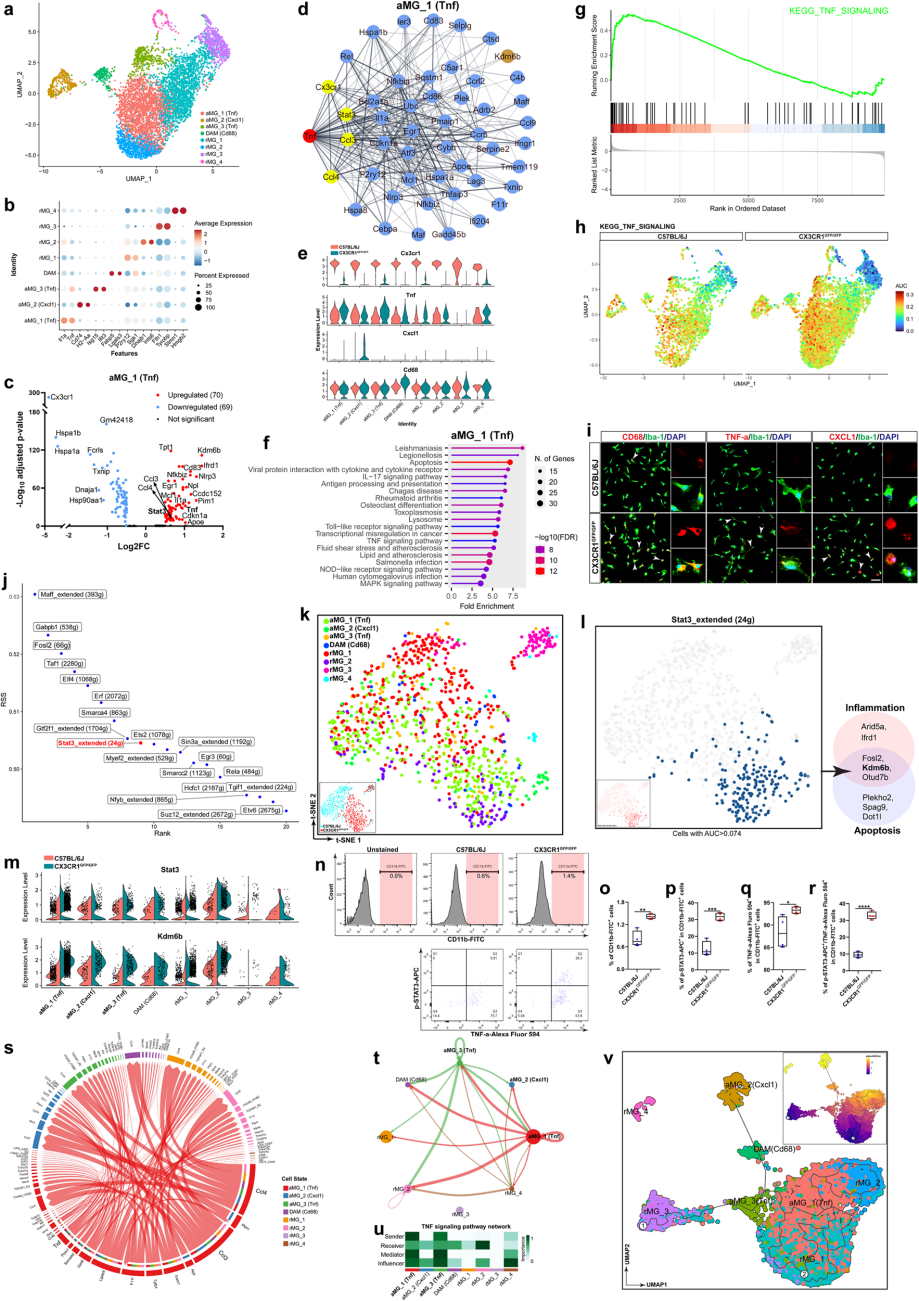
*Tnf*-dominant microglia contribute to increases in microglial STAT3 levels and neurotoxicity in CX3CR1-deficient retinas. **a**, UMAP plot showing eight unique microglial clusters from CX3CR1^GFP/GFP^ and C57BL/6J retinas at 6 weeks of age. **b**, Bubble chart showing marker gene expression in each cluster. **c**, Volcano plot showing DEGs in cluster aMG_1 (*Tnf* dominant). The red dots indicate upregulated DEGs (FC >1.5), and the blue dots indicate downregulated DEGs (FC <1.5). **d**, PPI network of DEGs in cluster aMG_1 (*Tnf* dominant). **e**, Violin plots showing *Cx3cr1*, *Tnf*, *Cd68* and *Cxcl1* expression in each cluster. **f**, KEGG pathway analysis of DEGs in aMG_1 (*Tnf* dominant). **g**, TNF signaling pathway in aMG_1 (*Tnf* dominant) by GSEA. **h**, UMAP plots showing the AUC activities of TNF signaling in microglia from CX3CR1^GFP/GFP^ and C57BL/6J retinas. **i,** Colocalization of Iba-1 with CD68, TNF-α or CXCL1 in MACS-sorted microglia from CX3CR1^GFP/GFP^ and C57BL/6J retinas at 6 weeks of age. Scale bar, 20 µm. **j**, Activity of the top 20 TFs in 1,000 microglia from CX3CR1^GFP/GFP^ and C57BL/6J retinas at 6 weeks of age. **k**, Distribution of 8 different clusters among 1,000 microglia from CX3CR1^GFP/GFP^ and C57BL/6J retinas. **l**, Extended regulon activity of *Stat3* among 1,000 microglia. **m**, *Stat3* and *Kdm6b* expression in each cluster. **n**–**r**, Flow cytometry analysis (**n**) and quantification of CD11b-FITC^+^ (**o**) and p-STAT3-APC^+^ (**p**), TNF-α-Alexa Fluor-594^+^ (**q**) and p-STAT3-APC^+/^TNF-α-Alexa Fluor-594^+^ (**r**) cells among CD11b^+^ microglia from CX3CR1^GFP/GFP^ and C57BL/6J retinas at 6 weeks of age (*n* = 4 mice per group). The data are presented as the mean ± s.e.m. and were analyzed via unpaired two-tailed Student’s *t*-tests (*P < 0.05, **P < 0.01, ***P < 0.001, ****P < 0.0001). **s**, LR interaction network among eight different clusters. **t**, Circle plot showing TNF signaling networks. **u**, Heatmap showing the relative importance of each cluster on the basis of the computed four network centrality measures of TNF signaling. **v**, Pseudotime trajectory analysis of microglia.

Previous studies have shown that microglia are composed of heterogeneous populations that perform diverse functions in various neurodegenerative diseases. To further elucidate how CX3CR1-deficient microglia contribute to increases in STAT3 levels, we performed scRNA-seq on microglia. In total, the RNA of 6313 microglia sorted from CX3CR1-deficient and C57BL/6J retinas was sequenced (Fig. 7a and Supplementary Fig. 4a). The data revealed high heterogeneity in CX3CR1-deficient microglia, making it possible to define eight transcriptional states on the basis of molecular signatures and functions (Fig. 7a, b). The classic homeostatic markers *Tmem119*, *P2ry12*, *Csf1r*, *C1qa* and *C1qb* were highly and widely expressed among the eight microglial clusters (Supplementary Fig. 4b–f), whereas the monocyte markers *Ly6c2* and *Ccr2* were barely detectable (Supplementary Fig. 4g, h). On the basis of the marker gene expression in each cluster, we defined three activated microglial clusters (aMG_1, aMG_2 and aMG_3) that expressed high levels of unique signature genes of activated microglia, including *Il1a*, *Tnf*, *H2-Aa*, *Cd74*, *Ifit3* and *Isg15*, and one cluster of disease-associated microglia (DAM) that expressed high levels of DAM marker genes, including *Fabp5* and *Lgals3* (Fig. 7b). PPI and violin plot analyses of the DEGs further revealed two *Tnf*-dominant microglial clusters, namely, aMG_1 and aMG_3; one *Cxcl1*-dominant cluster (aMG_2); and one *Cd68*-dominant cluster (DAM) (Fig. 7c–e and Supplementary Fig. 4i, j). *Tnf* strongly interacted with most of the DEGs in aMG_1 and aMG_3 microglia (Fig. 7d and Supplementary Fig. 4i), suggesting that the aMG_1 and aMG_3 clusters are *Tnf* dominant. In addition, *Cxcl1* was specifically expressed in aMG_2 microglia (Fig. 7e), suggesting that aMG_2 is *Cxcl1* dominant. Moreover, we found that *Cd68* expression was significantly increased (Fig. 7e) and that *Cd68* strongly interacted with most of the DEGs in the DAM cluster (Supplementary Fig. 4j), indicating that DAM is *CD68* dominant. KEGG pathway analysis revealed that DEGs in aMG_1 (*Tnf-*dominant), aMG_2 (*Cxcl1-*dominant) and aMG_3 (*Tnf-*dominant) microglia were associated mainly with inflammation, phagosomes and apoptosis (Fig. 7f and Supplementary Fig. 4k, l) and that DEGs in DAM (*Cd68* dominant) were involved mainly in degenerative processes (Supplementary Fig. 4). Indeed, we observed that the TNF signaling pathway, the Toll-like receptor signaling pathway and the apoptosis pathway were significantly enriched in aMG_1 (*Tnf*-dominant), aMG_2 (*Cxcl1*-dominant) and aMG_3 (*Tnf-*dominant) microglia (Fig. 7g,h and Supplementary Fig. 4n, o), whereas the degeneration pathway was increasingly upregulated in DAM (*Cd68*-dominant) microglia (Supplementary Fig. 4m, p). Consistently, immunostaining revealed that TNF-α, CD68 and CXCL1 were significantly upregulated in microglia purified from CX3CR1-deficient retinas compared with those from C57BL/6J retinas (Fig. 7i). Taken together, these data indicate that microglial CX3CR1 deficiency creates transcriptional heterogeneity in microglia.

Furthermore, we performed SCENIC analysis and revealed the top 20 TFs, including *Stat3*, in microglia from CX3CR1-deficient retinas (Fig. 7j). We also found that *Stat3* signaling was significantly upregulated in the CX3CR1-deficient microglia compared with the C57BL/6J microglia (Fig. 7k–m). In particular, we observed that *Stat3* enrichment occurred mainly in *Tnf*-dominant (aMG_1 and aMG_3) microglia (Fig. 7m).

Similarly, the percentage of p-STAT3^+^/TNF-α^+^ microglia was significantly greater (Fig. 7n–r) in CX3CR1-deficient retinas than in C57BL/6J retinas. However, there were no significant changes in the percentage of p-STAT3^+^/CXCL1^+^ or p-STAT3^+^/CD68^+^ microglia in CX3CR1-deficient retinas (Supplementary Fig. 4q–z). Together, these data indicate that *Tnf*-dominant microglia strongly contribute to STAT3 upregulation in the context of CX3CR1 deficiency.

By further analysis of the activity of the *Stat3* regulon in each cell, we identified 24 potential downstream targets of *Stat3* (Fig. 7k, l), including several factors that have been reported to regulate inflammation and apoptosis, such as *Arid5a*, *Ifrd1*, *Fosl2*, *Kdm6b*, *Otud7b*, *Plekho2*, *Spag9* and *Dot1l*^49–53^. Notably, we found that lysine (K)-specific demethylase 6B (*Kdm6b*) was consistently upregulated in conjunction with *Stat3* (Fig. 7m), which is consistent with the proteomic analysis. Together, these data suggest that CX3CR1-deficient microglia might mediate neurotoxicity via the *Tnf*/*Stat3*/*Kdm6b* signaling pathway.

CellChat analysis revealed that aMG_1 (*Tnf*-dominant) microglia engaged in intensive communication with other microglial clusters, including aMG_2 (*Cxcl1*-dominant), aMG_3 (*Tnf*-dominant), DAM (*Cd68*-dominant) and rMG_2 microglia, via various ligand‒receptor (LR) pairs, including *Ccl3*/*Ccr5*, *Ccl4*/*Ccr5*, *Tnf*/*Tnfrsf1b*, *Tnf*/*Tnfrsf1a*, *App*/*Cd74* and *Ccl6*/*Ccr1* (Fig. 7s). Among these ligands, *Tnf*, *Ccl3* and *Ccl4* were significantly enriched in aMG_1 (*Tnf*-dominant) microglia in CX3CR1-deficient retinas (Fig. 7c). In addition, we found that the TNF signaling pathway, which was activated mainly in aMG_1 (*Tnf*-dominant) and aMG_3 (*Tnf*-dominant) microglia, potentially affected aMG_2 (*Cxcl1*-dominant), DAM (*Cd68*-dominant) and rMG_2 microglia (Fig. 7t–u). Together, these results indicate that these ligands, including *Tnf*, *Ccl3* and *Ccl4*, in *Tnf*-dominant microglia might play important roles in regulating cell‒cell communication among microglia.

Single-cell trajectory analysis with Monocle3 revealed that microglia undergo a distinct trajectory from the homeostatic state to the activated state (Fig. 7v). *Tnf*-dominant microglia might be derived from homeostatic microglia and subsequently transition to DAM (*Cd68*-dominant), followed by aMG_2 (*Cxcl1*-dominant) microglia (Fig. 7v), suggesting that *Tnf*-dominant microglia may act as gatekeepers in mediating cell‒cell communication.

Overall, these findings demonstrate that *Tnf*-dominant microglia are responsible for increased microglial STAT3 levels, neurotoxicity and cell‒cell communication following CX3CR1 deficiency.

### *Ackr1* is a critical molecule for microglia‒cone photoreceptor communication and selective cone cell apoptosis in CX3CR1-deficient retinas

**Fig. 8:**
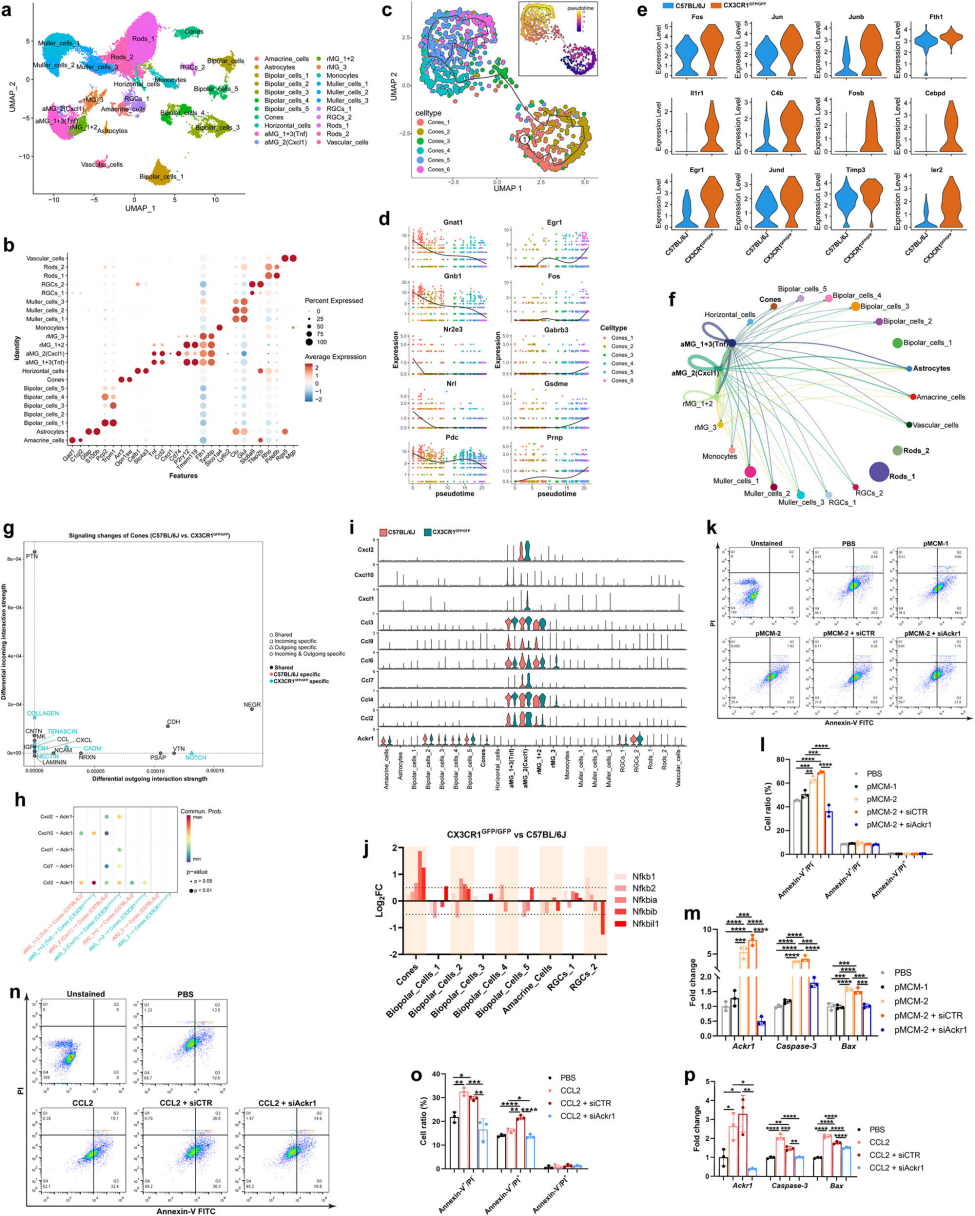
*Ackr1* is a critical molecule for microglia‒cone photoreceptor communication and selective cone-cell apoptosis in CX3CR1-deficient retinas. **a**, UMAP plot showing different retinal cell clusters from the retinas of 6-week-old CX3CR1^GFP/GFP^ and C57BL/6J mice. **b**, Marker gene expression in each cluster. **c**, Pseudotime analysis of six distinct cone cell clusters. **d**, Expression of phototransduction-associated genes (*Gnat*, *Gnb1*, *Nr2e3*, *Nrl* and *Pdc*) and apoptosis-associated genes (*Egr1*, *Fos*, *Gabrb3*, *Gsdme* and *Prnp*) in cones over pseudotime. **e**, Inflammation-related gene expression in astrocytes from the retinas of 6-week-old CX3CR1^GFP/GFP^ and C57BL/6J mice. **f**, Cell‒cell interactions between microglia and other retinal cells. **g**, Signaling changes in cones from the retinas of 6-week-old CX3CR1^GFP/GFP^ and C57BL/6J mice. **h**, LR interactions between microglia and cones. **i**, Violin plots showing CCL and CXCL signaling expression in different retinal cells. **j**, Expression of NF-ĸB signaling-associated DEGs in cones, bipolar cells, amines and RGCs between CX3CR1^GFP/GFP^ and C57BL/6J retinas. **k**, **l**, Flow cytometry analysis (**k**) and quantification (**l**) of the percentages of Annexin-V^+^/PI^−^, Annexin-V^+^/PI^+^ and Annexin-V^−^/PI^+^ cells among 661W cells pretreated with siAckr1 or siCTR and cocultured with pMCM-2 or pMCM-1. **m**, qPCR analysis of *Ackr1*, *Caspase-3* and *Bax* expression in 661W cells treated as described in **k**. **n**, **o**, Flow cytometry analysis (**n**) and quantification (**o**) of the percentages of Annexin-V^+^/PI^−^, Annexin-V^+^/PI^+^ and Annexin-V^−^/PI^+^ cells among 661W cells treated with CCL2 (100 ng/ml) after pretreatment with siAckr1 or siCTR. **p**, qPCR analysis of *Ackr1*, *Caspase-3* and *Bax* in 661W cells treated as described in **n**. The results represent three independent experiments. The data are presented as the mean ± s.e.m. and were analyzed via one-way ANOVA with Tukey’s multiple comparison test (*P < 0.05, **P < 0.01, ***P < 0.001, ****P < 0.0001).

To further elucidate how CX3CR1-deficient microglia mediate astrocyte reactivity and selective cone cell loss, we next performed scRNA-seq on whole CX3CR1-deficient and C57BL/6J retinas. To better explore the cell‒cell interactions between microglia and other retinal cells, we integrated microglial scRNA-seq data with whole retinal scRNA-seq data for an in-depth analysis (Supplementary Fig. 5a). In total, 44,347 cells from CX3CR1-deficient and C57BL/6J retinas were categorized into 22 cell clusters (Fig. 8a). According to the expression of canonical marker genes, we identified two clusters as rods, four clusters as microglia, one cluster as cones and one cluster as astrocytes (Fig. 8a, b). Two rod photoreceptor clusters (Rods_1 and Rods_2) specifically expressed the rod signature genes *Rho* and *Pde6b*, and one cone photoreceptor cluster (Cones) was characterized by the unique expression of the cone marker genes *Opn1sw* and *Arr3* (Fig. 8a, b). Moreover, we observed that the canonical astrocyte marker genes *Gfap* and *S100b* were highly expressed in the astrocyte cluster (Astrocytes) and that microglial marker genes, including *Tyrobp*, *Tmem119*, *P2ry12* and *Fth1*, were uniquely expressed in four microglial clusters (aMG_1 + 3 (*Tnf*-dominant), aMG_2 (*Cxcl1*-dominant), rMG_1 + 2 and rMG_3) (Fig. 8a, b).

Next, we analyzed the transcriptional changes in cones and astrocytes. The cones of CX3CR1-deficient retinas exhibited significant downregulation of phototransduction-associated genes (such as *Pdc*, *Gnat1*, *Gnb1* and *Cnga1*) but upregulation of apoptosis-associated genes (such as *Egr1*, *Fos*, *Tmsb10* and *Anxa5*) compared with those of C57BL/6J retinas (Supplementary Fig. 5b, c). Similarly, cell trajectory analysis of cones revealed that the levels of *Gnat1*, *Gnb1*, *Nr2e3*, *Nrl* and *Pdc*, which are involved mainly in maintaining visual function^54,55^, were decreased in cones (Fig. 8c, d). Conversely, the expression of cell apoptosis-related genes, including *Fos*, *Egr1*, *Gabrb3*, *Gsdme* and *Prnp*, constantly increased with pseudotime in cones (Fig. 8c, d). These data suggest that cone photoreceptor apoptosis occurs in CX3CR1-deficient retinas, which is consistent with our in vivo observations. Moreover, we observed significant increases in inflammation- and apoptosis-related signaling pathways and genes, including *Fos*, *Jun*, *Junb*, *Fth1*, *Il1r1*, *C4b*, *Fosb*, *Cebpd*, *Egr1*, *Jund*, *Timp3* and *Ier2*, in astrocytes from CX3CR1-deficient retinas (Fig. 8e and Supplementary Fig. 5d, e), indicating astrocyte reactivity, which is consistent with our in vivo findings.

Furthermore, we evaluated cell‒cell communication between microglia and other retinal cells, including cone and rod cells, via CellChat analysis. We found that *Tnf*-dominant (aMG_1 + 3: aMG_1 and aMG_3) and *Cxcl1*-dominant (aMG_2) microglia strongly interacted with cones but not rods (Fig. 8f). In addition, we observed more significant signaling changes (such as CCL and CXCL signaling) in cones (Fig. 8g) but not in rods in CX3CR1-deficient retinas (Supplementary Fig. 5f). Together, these data suggest that microglial CX3CR1 deficiency selectively targets cones but spares rods, which is consistent with our in vivo observations.

Analysis of the LR interactions between microglia and cones revealed that microglia-derived CCLs and CXCLs strongly interact with their receptor, atypical chemokine receptor 1 (*Ackr1*), in cones. We found that *Ackr1* was ubiquitously expressed in cones and other retinal cells (such as bipolar cells, amacrine cells and retinal ganglion cells (RGCs)) but was primarily upregulated in cones of CX3CR1-deficient retinas (Fig. 8h, i); these results suggest that *Ackr1* might be a critical molecule in regulating cone apoptosis in CX3CR1-deficient retinas. To understand the mechanism underlying *Ackr1* upregulation selectively in cones, we examined DEGs in cones and other retinal cells and found that NF-κB signaling-related DEGs were significantly upregulated in cones but not in other retinal cells in the context of CX3CR1 deficiency (Fig. 8j); this result is consistent with the expression pattern of *Ackr1*, suggesting that NF-κB signaling might induce *Ackr1* expression in cones. Similarly, a previous study reported that NF-κB signaling mediated *Ackr1* induction and expression^56,57^.

To verify the role of *Ackr1* in cone cell apoptosis, we cultured 661W cone cells that were pretreated with siAckr1 in pMCM-2 or pMCM-1 or cocultured siAckr1-treated 661W cone cells with recombinant CCL2, CCL3, CCL4 or CXCL12 proteins. We found that pMCM-2 treatment or coculture with one of the four recombinant chemokine proteins significantly increased 661W cone cell apoptosis compared with untreated or siCTR-treated control cells (Fig. 8k–p and Supplementary Fig. 5g–o). However, after *Ackr1* knockdown, we observed a markedly reduced number of Annexin-V^+^/PI^−^ cells and expression levels of proapoptosis-associated genes in 661W cone cells in response to treatment with pMCM-2 or one of the recombinant chemokine proteins (Fig. 8k–p and Supplementary Fig. 5g–o). Together, these data indicate that *Ackr1* is a critical molecule in the regulation of microglia-cone communication and selective cone cell apoptosis. Consistently, these neuroprotective phenotypes were recapitulated in vivo. Notably, knockdown of *Ackr1* in CX3CR1^GFP/GFP^ retinas via intravitreal injection with siACKR1 significantly inhibited microglial hyperactivation and cone photoreceptor degeneration (Supplementary Fig. 6a–c), further mechanistically linking chemokine signaling to the selective cone photoreceptor apoptosis.

### *Cxcl1*-dominant microglia contribute to microglia‒astrocyte communication and increased STAT3 expression in astrocytes in CX3CR1-deficient retinas

**Fig. 9:**
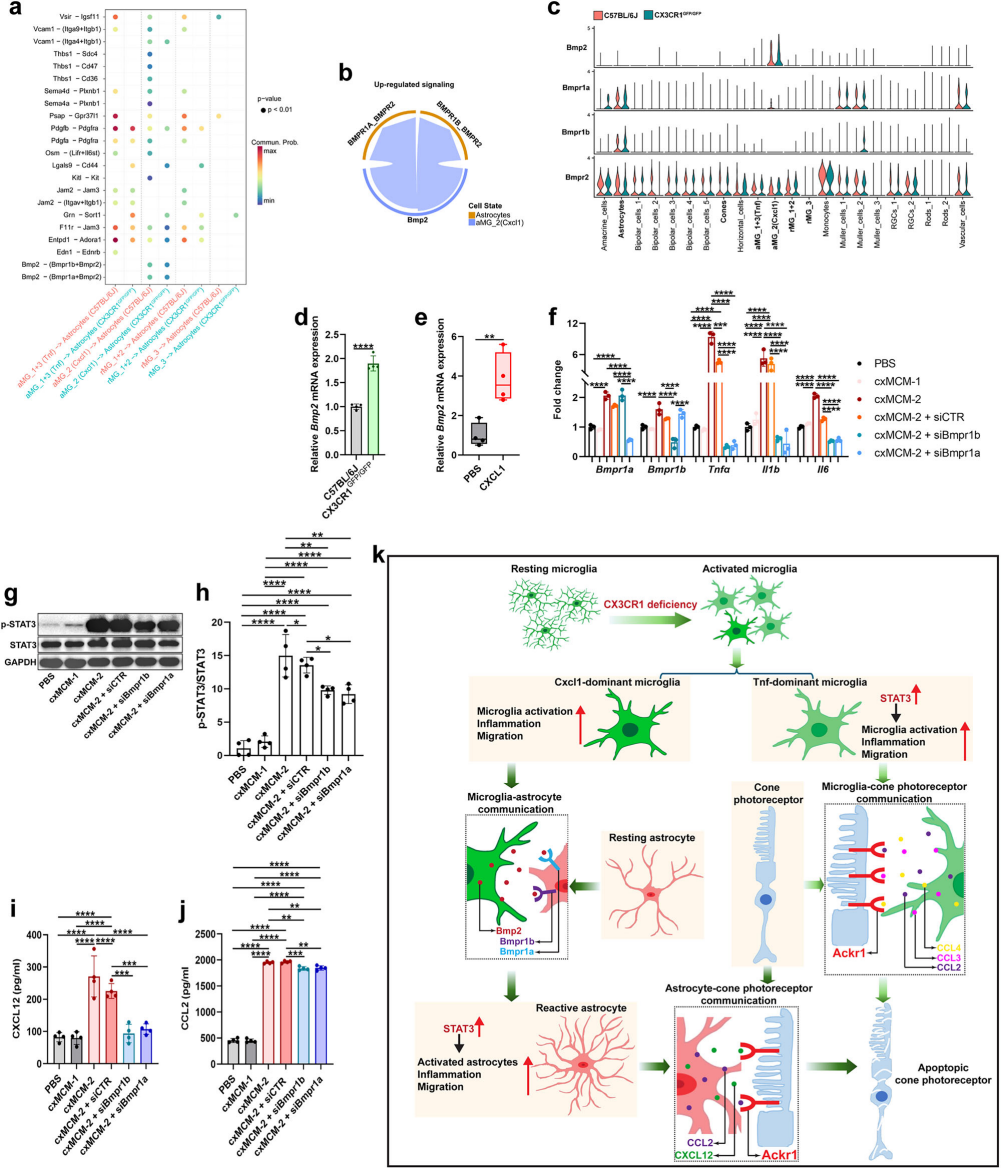
*Cxcl1*-dominant microglia contribute to increased STAT3 levels in astrocytes and microglia‒astrocyte communication in CX3CR1-deficient retinas. **a**, LR interactions between microglia and astrocytes. **b**, Upregulated signaling between microglia and astrocytes. **c**, *Bmp* signaling expression in different retinal cell clusters. **d**, *Bmp2* expression in MACS-sorted microglia from CX3CR1^GFP/GFP^ and C57BL/6J mouse retinas. **e**, *Bmp2* expression in BV2 cells following treatment with the recombinant CXCL1 protein (100 ng/ml)**. f**, qPCR analysis of *Bmpr1a*, *Bmpr1b*, *Tnfα*, *Il1b* and *Il6* expression in IMA2.1 cells cocultured with cxMCM-2 or cxMCM-1 after pretreatment with siBmpr1a, siBmpr1b or siCTR. **g**, **h**, Western blotting analysis (**g**) and quantification (**h**) of p-STAT3 and STAT3 expression in IMA2.1 cells cocultured with cxMCM-2 or cxMCM-1 after pretreatment with siBmpr1a, siBmpr1b or siCTR. **i**, **j**, ELISA analysis of CXCL12 (**i**) and CCL2 (**j**) expression in cell supernatants from IMA2.1 cells cocultured with cxMCM-2 or cxMCM-1 after pretreatment with siBmpr1a, siBmpr1b or siCTR. The results shown represent three to four independent experiments. The data are presented as the mean ± s.e.m. and were analyzed via one-way ANOVA with Tukey’s multiple comparison test or unpaired two-tailed Student’s *t*-test (*P < 0.05, **P < 0.01, ***P < 0.001, ****P < 0.0001). **k**, Schematic illustration showing the role of CX3CR1/STAT3/CCL–ACKR1 signaling in mediating the selective vulnerability of cone photoreceptors in the mouse retina.

To elucidate how CX3CR1-deficient microglia regulate astrocyte reactivity, we analyzed LR interactions between microglia and astrocytes. We found that multiple LR pairs, including *Bmp2*–*Bmpr1a*/*Bmpr1b*, *Edn1*–*Ednrb*, *F11r*–*Jam3* and *Lgals9*–*Cd44*, were involved in microglia‒astrocyte communication (Fig. 9a). Interestingly, we observed significant upregulation of *Bmp2* signaling in *Cxcl1*-dominant microglia (aMG_2) and of *Bmpr1a* and *Bmpr1b* signaling in astrocytes (Fig. 9b, c), indicating the potential involvement of the *Bmp2*–*Bmpr1a*/*Bmpr1b* pair in microglia‒astrocyte communication in the context of CX3CR1 deficiency. Consistently, we detected significant *Bmp2* upregulation in CX3CR1-deficient microglia in vivo and in BV2 microglia in vitro after treatment with the recombinant CXCL1 protein (Fig. 9d, e).

For our next analysis, we cultured IMA2.1 astrocytes that were pretreated with siCTR, siBmpr1a or siBmpr1b in culture medium from BV2 microglia that were pretreated with the recombinant CXCL1 protein (cxMCM-2) or PBS (cxMCM-1). We found that cxMCM-2 treatment significantly upregulated *Bmpr1a*, *Bmpr1b* and proinflammatory molecules in IMA2.1 astrocytes (Fig. 9f); however, these effects were largely reversed in IMA2.1 astrocytes after *Bmpr1a* or *Bmpr1b* knockdown (Fig. 9f). In addition, we found that cxMCM-2 treatment significantly upregulated p-STAT3 in IMA2.1 cells and that this upregulation was reduced after *Bmpr1a* or *Bmpr1b* knockdown (Fig. 9g, h), suggesting that *Bmp2*–*Bmpr1a*/*Bmpr1b* signaling regulates STAT3 in astrocytes. Notably, cxMCM-2 treatment significantly increased CCL2 and CXCL12 production in IMA2.1 cells, which was also suppressed by *Bmpr1a* or *Bmpr1b* knockdown (Fig. 9I, j). Taken together, these data suggest that *Cxcl1*-dominant microglia communicate with astrocytes via the *Bmp2*–*Bmpr1a*/*Bmpr1b* pair, contributing to the increases in STAT3, CCL2 and CXCL12 expression in astrocytes.

In conclusion, we demonstrate that microglial CX3CR1 deficiency results in transcriptional heterogeneity in microglia, producing multiple subpopulations of microglia with unique molecular and functional characteristics. Among them, *Tnf*-dominant microglia contribute to increased STAT3 expression in microglia and the subsequent release of CCL chemokines that interact with their receptor *Ackr1*, which is upregulated primarily in cone cells, ultimately leading to selective cone cell vulnerability (Fig. 9k). Moreover, *Cxcl1*-dominant microglia communicate with astrocytes via the *Bmp2*–*Bmpr1a*/*Bmpr1b* pair, triggering STAT3 upregulation and astrocyte reactivity. Reactive astrocytes then secrete the toxic chemokines CCL2 and CXCL12, which bind to their receptor *Ackr1* in cone cells, resulting in additional cone cell death (Fig. 9k). Thus, targeting CX3CR1/STAT3 signaling could be a therapeutic strategy to combat microglial neurotoxicity.

## Discussion

In this study, we used the retina, which is known to be an extension of the brain and shares many characteristics with the most common neurodegenerative disorders, as a model of the brain to investigate the cellular and molecular mechanisms underlying the selective neuronal vulnerability that occurs in different neurodegenerative diseases, including AD^58,59^. We discovered that disruption of CX3CR1 signaling in microglia induced proinflammatory responses in microglia and then astrocytes via the upregulation of STAT3 signaling, leading to the selective apoptosis of cone photoreceptors in the mouse retina. Mechanistically, we demonstrated that CX3CR1 deficiency induced distinct transcriptional heterogeneity in microglia and identified two subpopulations of microglia that are responsible for this activity. Specifically, we found that *Tnf*-dominant microglia communicate mainly with cone photoreceptors through CCLs and their receptor *Ackr1*; *Ackr1* expression is upregulated primarily in cones through NF-κB signaling, which is markedly upregulated in cones but not in other retinal cells in the context of CX3CR1 deficiency, ultimately leading to selective cone vulnerability. Moreover, *Cxcl1*-dominant microglia communicate with astrocytes primarily via *Bmp2*–*Bmpr1a*/*Bmpr1b* signaling to upregulate STAT3 and activate astrocytes, which then interact with cone photoreceptors through the CCL/CXCL–ACKR1 pair, ultimately leading to additional cone cell death. Our findings suggest that cell‒cell communication between neurons and microglia could be critical for triggering selective neuronal vulnerability in different contexts of neurodegenerative diseases.

CX3CR1, which is an important cell surface receptor on microglia in the brain, modulates microglial homeostasis, migration, phagocytosis and inflammation in neurodegenerative diseases, including AD, multiple sclerosis, spinal cord injury and traumatic brain injury^5,10,60–64^. Emerging evidence demonstrates that CX3CR1 signaling disruption exacerbates neuronal degeneration and cognitive deficits in both AD and amyotrophic lateral sclerosis models, underscoring its essential role in neuronal survival maintenance^5,65,66^. Our findings extend these observations to retinal pathophysiology, revealing that constitutive CX3CR1 activity sustains cone photoreceptor integrity in the retinas, aligning with previous reports of CX3CR1’s essential role in maintaining retinal homeostasis^14,15,67^. However, in ischemic stroke models, CX3CR1 deficiency attenuates microglial activation and neuronal apoptosis by blocking calcium influx and reactive oxygen species generation^68,69^. These functional divergences of microglial CX3CR1 in different disease models probably stem from distinct microenvironmental signatures (chronic versus acute inflammation, cell-type-specific interactomes) rather than intrinsic receptor duality. Future lineage-tracing studies comparing CX3CR1^+^ microglial dynamics across inflammatory paradigms should be warranted to systematically test how different neuroinflammatory contexts rewire CX3CR1-mediated neuron–glia interactions.

STAT3, which is a member of the STAT family of cytoplasmic TFs, plays a vital role in transmitting extracellular signals from cell surface receptors to the nucleus in response to various stimuli, including inflammatory cytokines and growth factors^70,71^. Our in vivo and in vitro data demonstrated that STAT3 could be a potential downstream target of CX3CR1 that contributes to microglial activation and astrocyte reactivity. We revealed that *Tnf*-dominant microglia contribute mainly to STAT3 upregulation in microglia and microglial activation, whereas *Cxcl1*-dominant microglia trigger STAT3 upregulation in astrocytes and astrocyte reactivity through microglia‒astrocyte communication. Consistent with our findings, STAT3 activation in microglia reportedly promotes proinflammatory cytokine expression and neuronal apoptosis in various brain diseases^72,73^. Similarly, STAT3 upregulation in astrocytes results in reactive astrocytes, inflammation and neuronal death in neurodegenerative diseases^74–76^. Meanwhile, we observed that genetic inhibition of STAT3 signaling significantly ameliorated microglial activation, astrocyte reactivity and cone photoreceptor loss in CX3CR1-deficient retinas, confirming the detrimental effects of STAT3.

As a typical receptor of chemokine signals, *Ackr1* is widely expressed in erythroid cells, endothelial cells and neurons in the central nervous system^77^, and it regulates the proinflammatory response, cell apoptosis and proliferation in multiple disease contexts^78,79^. Here, we demonstrated that *Ackr1* was selectively upregulated in cones, and this upregulation was mediated by increased NF-κB signaling primarily in cones but not in other retinal cells after CX3CR1 deficiency, subsequently resulting in the selective vulnerability of cone cells. Our subsequent in vitro study further verified that *Ackr1* activation increased proapoptotic signaling in 661W cone photoreceptor cells, whereas genetic knockdown of *Ackr1* markedly ameliorated cone cell death, suggesting the detrimental effect of *Ackr1* on cone cell survival. Although the mechanism by which *Ackr1* induces cone apoptosis remains to be elucidated, our findings suggest that targeting ACKR1 signaling could be a promising therapeutic strategy for improving cone survival and function, which could be beneficial for a wide range of patients with retinitis pigmentosa with degeneration of cone photoreceptors in the retina and potentially for other patients with photoreceptor degeneration that affects vision, such as age-related macular degeneration, which is the leading cause of blindness worldwide. Further investigations are warranted to explore the role of ACKR1 in regulating cone photoreceptor death in the context of retinal degenerative diseases in the future.

Our study markedly advances the understanding of CX3CR1-dependent neuroimmune crosstalk in selective neuronal vulnerability, offering novel mechanistic insights distinct from prior research on retinal neuroinflammation. Unlike earlier studies that primarily focused on CX3CR1’s role in dampening microglial activation or its broad association with neurodegeneration^5,13^, we unveil a hierarchical cell‒cell communication network involving microglial subpopulation heterogeneity, CX3CR1/STAT3 signaling crosstalk and neuron–glia interactions that drive context-dependent neuronal loss. We identified two functionally divergent microglial subpopulations (*Tnf* dominant and *Cxcl1* dominant) in the context of CX3CR1 deficiency, a finding not reported in prior retinal or brain studies. These subpopulations mediate distinct pathways to target cone photoreceptors and astrocytes, respectively, explaining the selectivity of neuronal vulnerability. Moreover, we delineated a feedforward loop where *Cxcl1*-dominant microglia activate astrocytes via *Bmp2*–*Bmpr1a*/*Bmpr1b* signaling, which subsequently amplifies STAT3-dependent neurotoxicity through CCL/CXCL–ACKR1 interactions. This triad highlights astrocytes as active contributors to selective neurodegeneration, beyond their classical supportive roles. In addition, using single-cell transcriptomic profiling and morphology observation, we excluded the potential involvement of Müller cells in regulating cone-specific degeneration in this model (Supplementary Fig. 7).

In summary, our work provides a comprehensive understanding of how disrupted CX3CR1 signaling regulates selective neuronal vulnerability, suggesting potential novel therapeutic strategies that directly target the mechanisms underlying vulnerability or strategies that promote the mechanisms underlying resilience in vulnerable neurons in neurological disorders. Targeting CX3CR1/STAT3 signaling could be a critical therapeutic strategy for combating neurological disorders involving microglial neurotoxicity.

## Availability of data and materials

All data generated in this study are included in this article. Data and materials are available to any interested parties upon request.

## Supplementary information

Supplementary Information
