# Supplementary material for: Microglial CX3CR1 deficiency regulates the selective vulnerability of cone photoreceptors via STAT3/CCL–ACKR1 signaling in the mouse retina
Source: Exp Mol Med. 2026 Jan 15;58(1):178–98. doi: 10.1038/s12276-025-01618-7 (PMC12868684; doi:10.1038/s12276-025-01618-7)
Supplement: Supplementary file 1 — Supplementary Information [file 12276_2025_1618_MOESM1_ESM.pdf]

Supplementary Information

Supplementary Figures 1-7

Supplementary Table 1

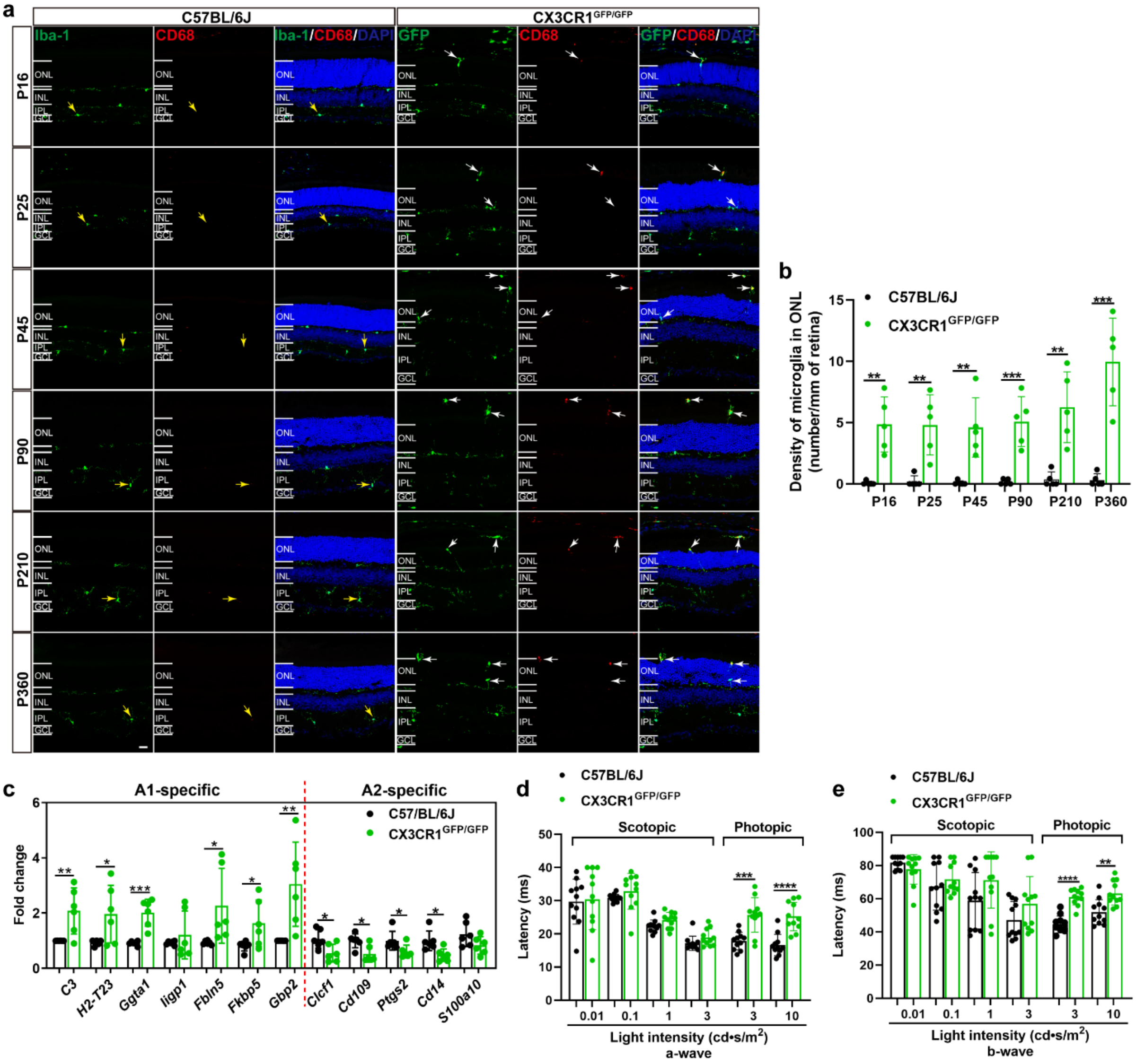

Supplementary Fig. 1: Microglial CX3CR1 deficiency induces glial reactivity and the selective vulnerability of cone photoreceptors in the retina. a, Retinal sections from CX3CR1$^{GFP/GFP}$ and C57BL/6J mice at P16, P25, P45, P90, P210 and P360 were stained with anti-CD68 and anti-Iba-1 antibodies. The yellow arrows indicate the resting microglial cell, and the white arrows indicate the dendritic migration of activated microglia into the ONL. **b,** Quantification of activated microglia in the ONL of retinas from CX3CR1$^{GFP/GFP}$ and age-matched C57BL/6J mice (n=5 mice/group). **c,** qPCR analysis of the A1-specific genes *C3*, *H2-T23*, *Ggta1*, *Iigp1*, *Fbln5*, *Fkbp5* and *Gbp2* and the A2-specific genes *Clcf1*, *Cd109*, *Ptgs2*, *Cd14* and *S100a10* in retinas from CX3CR1$^{GFP/GFP}$ and C57BL/6J mice (n=6 mice/group). **d-e** latency of ERG recordings under both scotopic and photopic conditions in 6-week-old CX3CR1$^{GFP/GFP}$ and C57BL/6J mice (n=11 mice/group). The data are presented as the means ± SEMs and were analysed via unpaired two-tailed Student's *t* tests (CX3CR1$^{GFP/GFP}$ vs. C57BL/6J, *p < 0.05, **p < 0.01, ***p < 0.001, ****p < 0.0001).

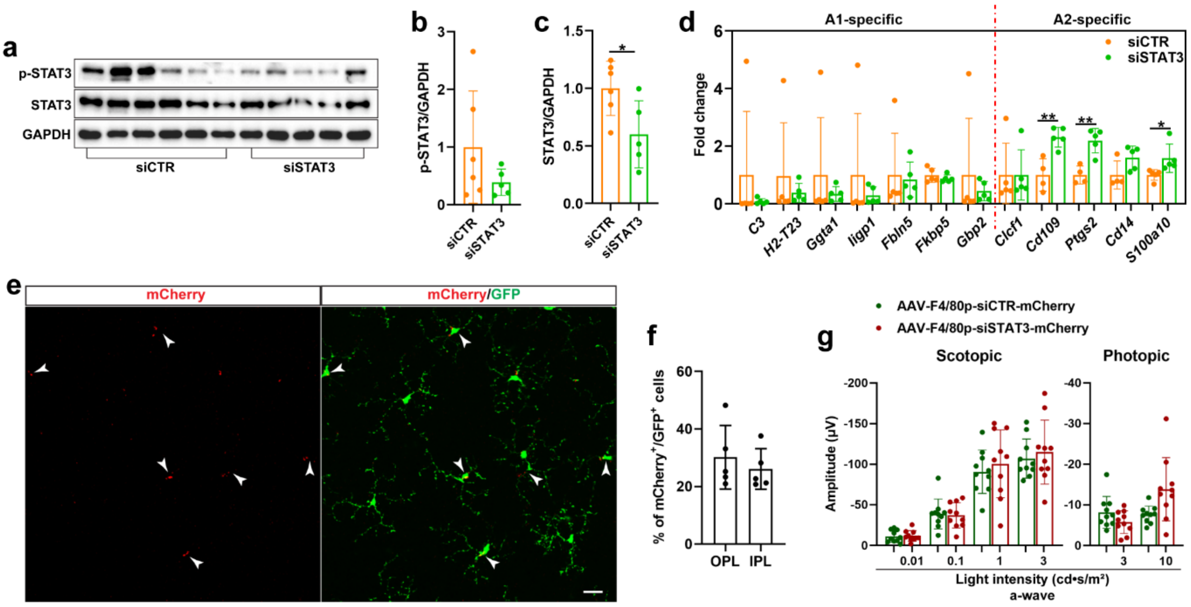

Supplementary Fig. 2: STAT3 knockdown ameliorates microglial neurotoxicity in CX3CR1-deficient retinas. a–c, Western blotting analysis (**a**) and quantification of p-STAT3 (**b**) and STAT3 (**c**) expression in 6-week-old CX3CR1$^{GFP/GFP}$ mice treated with siSTAT3 or siCTR (n=5–6 mice/group). **d,** qPCR analysis of A1- and A2-specific genes in the retinas of CX3CR1$^{GFP/GFP}$ mice treated with siSTAT3 or siCTR (n=4 mice/group). **e,** Colocalization of mCherry^{+} and GFP^{+} cells in retinal whole mounts from 6-week-old CX3CR1$^{GFP/GFP}$ mice treated with AAV-F4/80p-siSTAT3-mCherry. Scale bar, 20 µm. **f,** Quantification of the percentages of mCherry^{+}/GFP^{+} cells in the IPL and OPL of retinal whole mounts from CX3CR1$^{GFP/GFP}$ mice treated with AAV-F4/80p-siSTAT3-mCherry. **g,** ERG recordings of CX3CR1$^{GFP/GFP}$ mice at 6 weeks of age treated with AAV-F4/80p-siSTAT3-mCherry or AAV-F4/80p-siCTR-mCherry (n=10 mice/group). The data are presented as the means ± SEMs and were analysed via unpaired two-tailed Student's *t* tests (siSTAT3 vs. siCTR, *$p < 0.05$, **$p < 0.01$).

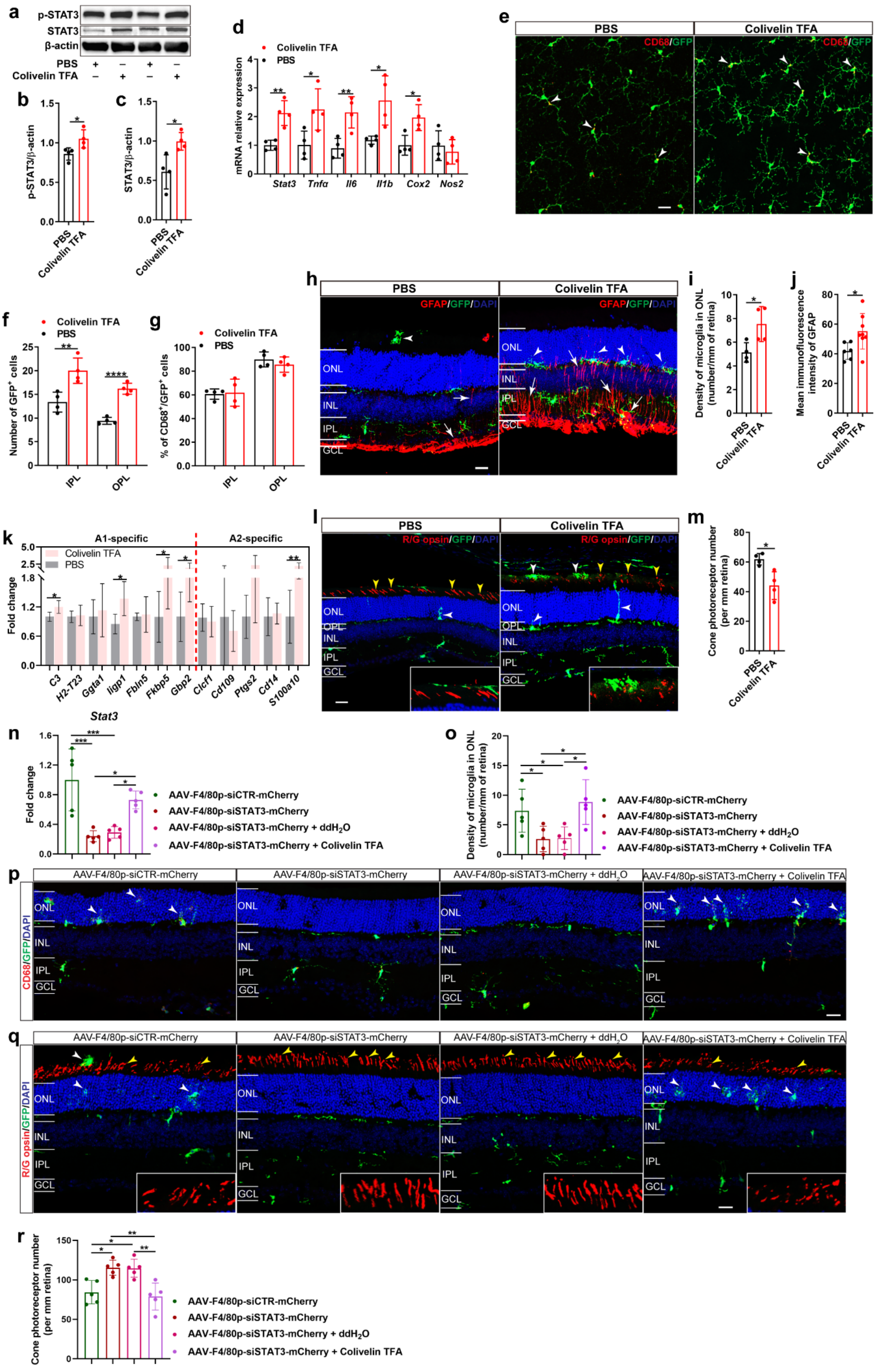
a
p-STAT3
STAT3
β-actin
PBS
Colivelin TFA
b
p-STAT3/β-actin
c
STAT3/β-actin
d
Colivelin TFA
PBS
mRNA relative expression
Stat3
Tnfα
Il6
Il1b
Cox2
Nos2
e
PBS
Colivelin TFA
CD68/GFP
f
Number of GFP+ cells
IPL
OPL
g
% of CD68+/GFP+ cells
h
GFAP/GFP/DAPI
ONL
INL
IPL
GCL
i
Density of microglia in ONL (number/mm of retina)
j
Mean immunofluorescence intensity of GFAP
k
A1-specific
A2-specific
Fold change
C3
H2-T23
Ggta1
Iigp1
Fbln5
Fkbp5
Gbp2
Clcf1
Cd109
Ptgs2
Cd14
S100a10
l
R/G opsin/GFP/DAPI
OPL
m
Cone photoreceptor number (per mm retina)
n
Stat3
AAV-F4/80p-siCTR-mCherry
AAV-F4/80p-siSTAT3-mCherry
AAV-F4/80p-siSTAT3-mCherry + ddH2O
AAV-F4/80p-siSTAT3-mCherry + Colivelin TFA
o
p
CD68/GFP/DAPI
q
R/G opsin/GFP/DAPI
r

Supplementary Fig. 3: Pharmacological activation of STAT3 exacerbates microglial neurotoxicity in CX3CR1-deficient retinas. a-c, Western blotting analysis (**a**) and quantification of p-STAT3 (**b**) and STAT3 (**c**) expression in the retinas of 6-week-old CX3CR1$^{GFP/GFP}$ mice treated with the STAT3 activator Colivelin TFA or PBS (n=4 mice/group). **d,** qPCR analysis of *Stat3* and proinflammatory molecule expression (n=4 mice/group). **e,** Retinal whole mounts from CX3CR1$^{GFP/GFP}$ mice stained for CD68. The white arrowheads indicate CD68-positive microglia. **f-g,** Quantification of GFP^{+} (**f**) and CD68^{+}/GFP^{+} microglia (**g**) in the retinas of CX3CR1$^{GFP/GFP}$ mice (n=4 mice/group). **h,** Retinal sections from CX3CR1$^{GFP/GFP}$ mice stained with GFAP. The white arrows indicate the extension of the dendritic processes of activated astrocytes into the IPL, and the white arrowheads indicate the dendritic migration of activated microglia into the ONL. **i,** Quantification of microglial cell density in the ONL of retinas from CX3CR1$^{GFP/GFP}$ mice (n=4 mice/group). **j,** Quantification of the mean immunofluorescence intensity of GFAP in (**h**) (n=6–8 mice/group). **k,** qPCR analysis of A1- and A2-specific gene expression in CX3CR1$^{GFP/GFP}$ retinas (n=4 mice/group). **l,** Retinal sections from CX3CR1$^{GFP/GFP}$ mice stained with an anti-R/G opsin antibody. Yellow arrowheads indicate cone photoreceptors. The boxed regions are highly magnified at the bottom. The white arrowheads show the dendritic migration of activated microglia into the ONL. **m,** Quantification of cone photoreceptors in (**l**) (n=4 mice/group). **n**, Validation of *Stat3* knockdown by qPCR (n=5 mice/group). **o**, Quantification of microglial density in the ONL of the retinas in (**p**) (n=5 mice/group). **p**, Retinal sections from 6-week-old CX3CR1$^{GFP/GFP}$ mice administrated with AAV-F4/80p-siSTAT3-mCherry and Colivelin TFA stained with CD68 antibody. White arrowheads show migration of activated microglia into the ONL. **q**, Retinal sections from 6-week-old CX3CR1$^{GFP/GFP}$ mice stained with R/G opsin antibody. White arrowheads show migration of activated microglia into the ONL, and yellow arrowheads indicate R/G opsin^{+} cone photoreceptors. The boxed regions are highly magnified at the bottom. **r**, Quantification of R/G opsin^{+} cone photoreceptors in (**q**) (n=5 mice/group). Scale bar, 20 µm. The data are presented as the means ± SEMs and were analysed via an unpaired two-tailed Student's *t* test or one-way ANOVA with Tukey’s multiple comparison test (Colivelin TFA vs. PBS, $*p < 0.05$, $**p < 0.01$, $****p < 0.0001$; compared to AAV-F4/80p-siSTAT3-mCherry, $p < 0.05$, $**p < 0.01$).

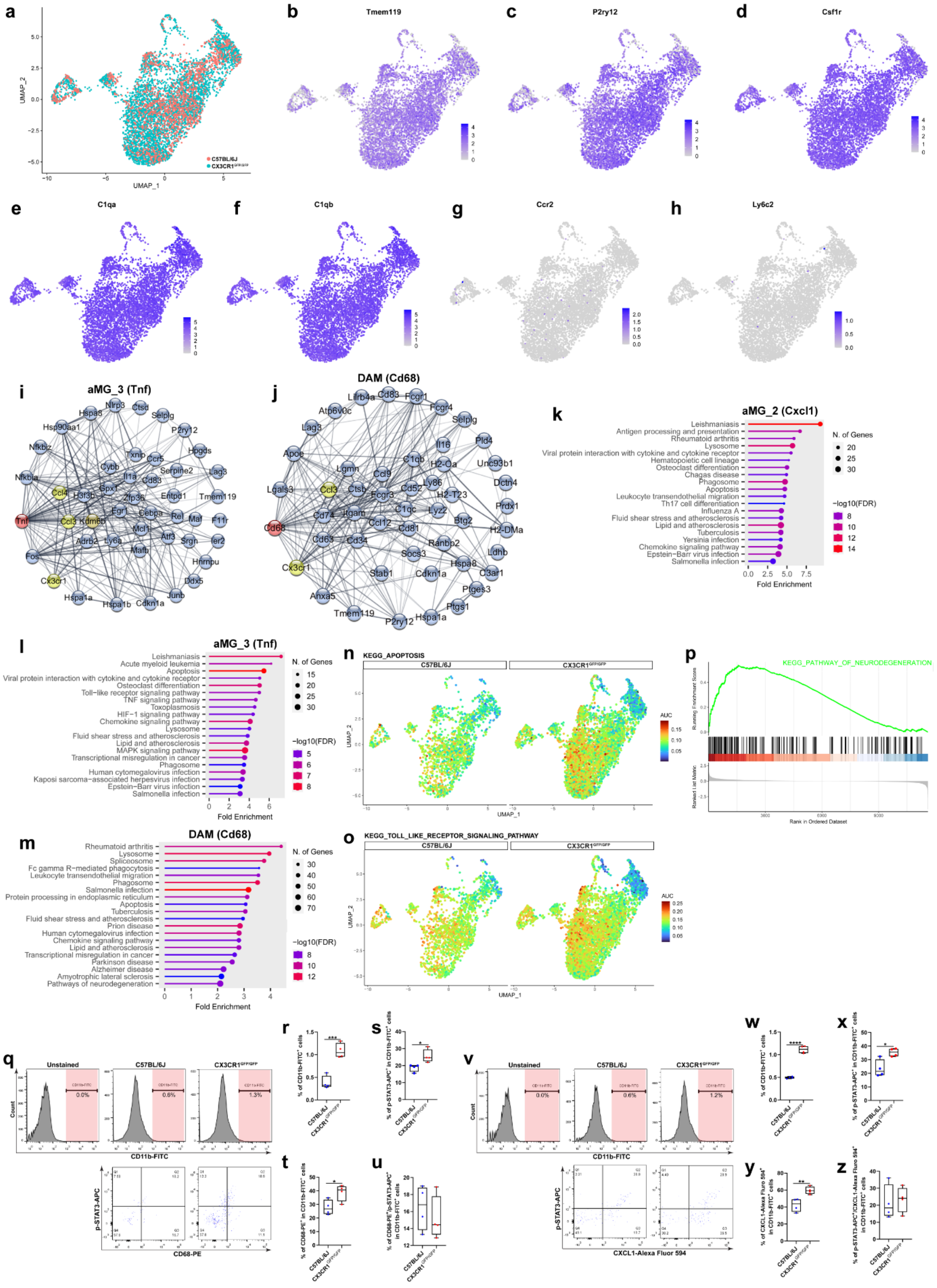

Supplementary Fig. 4: Microglial CX3CR1 deficiency triggers transcriptional changes in microglia. a, UMAP plots showing the distribution of different microglial clusters from CX3CR1$^{GFP/GFP}$ and C57BL/6J retinas of 6-week-old mice. **b-h,** UMAP plots showing *Tmem119* (**b**), *P2ry12* (**c**), *Csf1r* (**d**), *C1qa* (**e**), *C1qb* (**f**), *Ly6c2* (**g**) and *Ccr2* (**h**) expression

across all different microglial clusters from CX3CR1$^{GFP/GFP}$ and C57BL/6J retinas of 6-week-old mice. **i,** PPI network analysis of DEGs in aMG_3 (*Tnf*-dominant). **j,** PPI network analysis of DEGs in DAM (*Cd68*-dominant). **k-m,** KEGG pathway analysis of DEGs in aMG_2 (*Cxcl1*-dominant) (**k**), aMG_3 (*Tnf*-dominant) (**l**) and DAM (*Cd68*-dominant) (**m**). **n-o,** UMAP plots showing the AUC activities of the apoptosis (**n**) and Toll-like receptor signalling pathways (**o**) in each microglial cluster from the retinas of CX3CR1$^{GFP/GFP}$ and C57BL/6J mice at 6 weeks of age. **p,** Neurodegenerative pathway in DAM (*Cd68*-dominant) according to GSEA. **q-u,** Flow cytometry analysis (**q**) and quantification of percentages of CD11b-FITC^{+} (**r**) and p-STAT3-APC^{+} (**s**), CD68-PE^{+} (**t**), and p-STAT3-APC^{+}/CD68-PE^{+} (**u**) cells among CD11b-FITC^{+} microglia from the retinas of CX3CR1$^{GFP/GFP}$ and C57BL/6J mice at 6 weeks of age (n=4 mice). **v-z,** Flow cytometry analysis (**v**) and quantification of the percentages of CD11b-FITC^{+} (**w**) and p-STAT3-APC^{+} (**x**), CXCL1-Alexa Fluro-594^{+} (**y**) and p-STAT3-APC^{+}/CXCL1-Alexa Fluro-594^{+} (**z**) CD11b-FITC^{+} microglia in the retinas of 6-week-old (n=4 mice) CX3CR1$^{GFP/GFP}$ and C57BL/6J mice. The data are presented as the means ± SEMs and were analysed via an unpaired two-tailed Student's *t* test (CX3CR1$^{GFP/GFP}$ vs. C57BL/6J, *$p < 0.05$, **$p < 0.01$, ***$p < 0.001$, ****$p < 0.0001$).

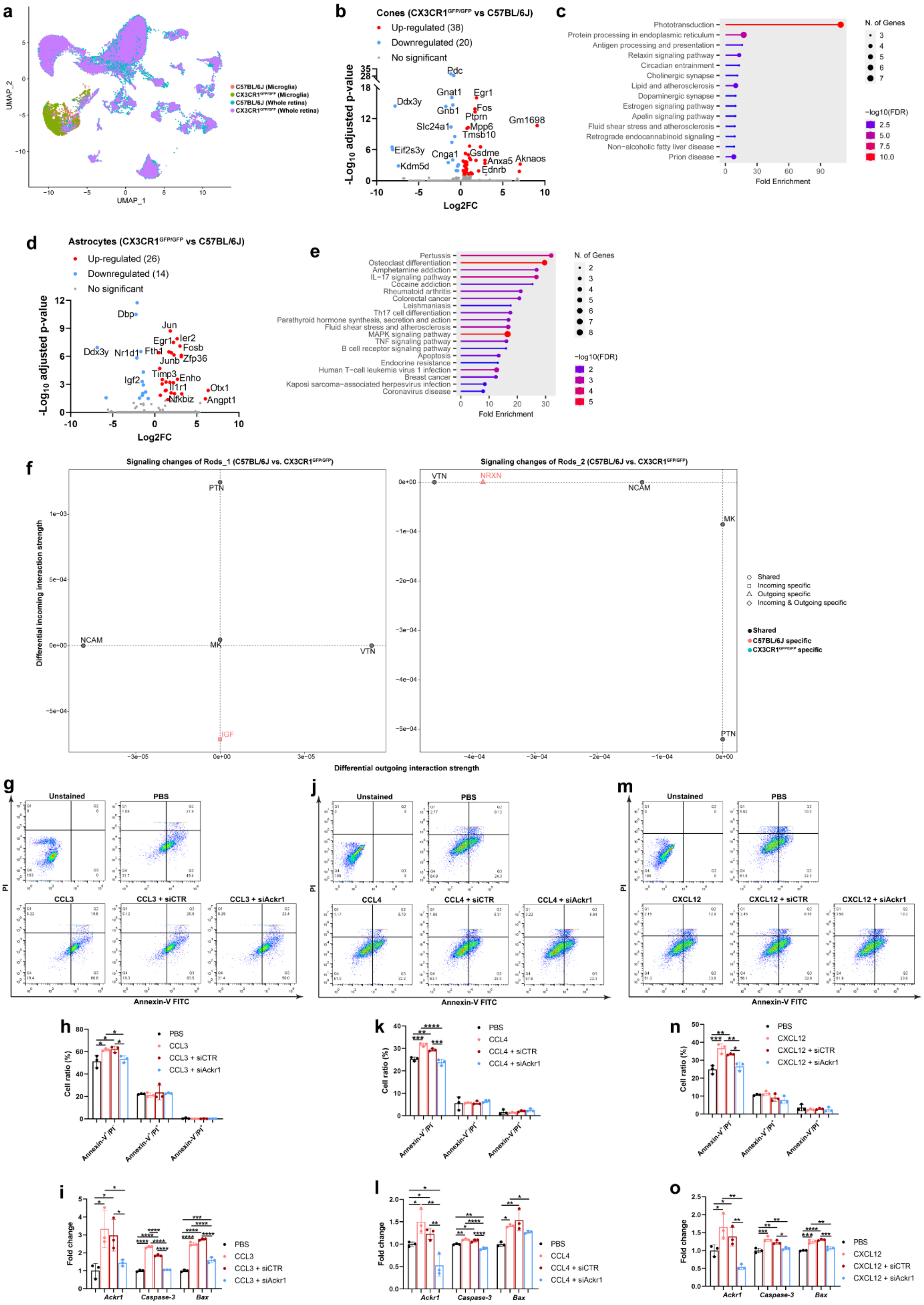

Supplementary Fig. 5: *Ackr1* is a critical molecule for microglia-cone photoreceptor communication and selective cone cell death in CX3CR1-deficient retinas. a, UMAP plot showing the distributions of microglia and other retinal cells from CX3CR1$^{GFP/GFP}$ and

C57BL/6J mice at 6 weeks of age. **b,** DEGs in cones between CX3CR1$^{GFP/GFP}$ and C57BL/6J mice. **c,** KEGG pathway analysis of DEGs in cones between CX3CR1$^{GFP/GFP}$ and C57BL/6J mice. **d,** DEGs in astrocytes between CX3CR1$^{GFP/GFP}$ and C57BL/6J mice. **e,** KEGG pathway analysis of DEGs in astrocytes between CX3CR1$^{GFP/GFP}$ and C57BL/6J mice. **f,** Signalling changes in rods between CX3CR1$^{GFP/GFP}$ and C57BL/6J retinas. **g-h,** Flow cytometry analysis (**g**) and quantification (**h**) of the percentages of Annexin-V^{+}/PI^{-}, Annexin-V^{+}/PI^{+} and Annexin-V^{-}/PI^{+} cells among 661W cells treated with CCL3 (100 ng/ml) after pretreatment with siAckr1 or siCTR. **i,** qPCR analysis of *Ackr1*, *Caspase-3* and *Bax* expression in 661W cells treated with CCL3 (100 ng/ml) after pretreatment with siAckr1 or siCTR. **j-k,** Flow cytometry analysis (**j**) and quantification (**k**) of the percentages of Annexin-V^{+}/PI^{-}, Annexin-V^{+}/PI^{+} and Annexin-V^{-}/PI^{+} cells among 661W cells treated with CCL4 (100 ng/ml) after pretreatment with siAckr1 or siCTR. **l,** qPCR analysis of *Ackr1*, *Caspase-3* and *Bax* expression in 661W cells treated with CCL4 (100 ng/ml) after pretreatment with siAckr1 or siCTR. **m-n,** Flow cytometry analysis (**m**) and quantification (**n**) of the percentages of Annexin-V^{+}/PI^{-}, Annexin-V^{+}/PI^{+} and Annexin-V^{-}/PI^{+} cells among 661W cells treated with CXCL12 (100 ng/ml) after pretreatment with siAckr1 or siCTR. **o,** qPCR analysis of *Ackr1*, *Caspase-3* and *Bax* expression in 661W cells treated with CXCL12 (100 ng/ml) after pretreatment with siAckr1 or siCTR. The results shown represent 3 independent experiments. The data are presented as the means ± SEMs and were analysed by one-way ANOVA with Tukey's multiple comparison test (*$p < 0.05$, **$p < 0.01$, ***$p < 0.001$, ****$p < 0.0001$).

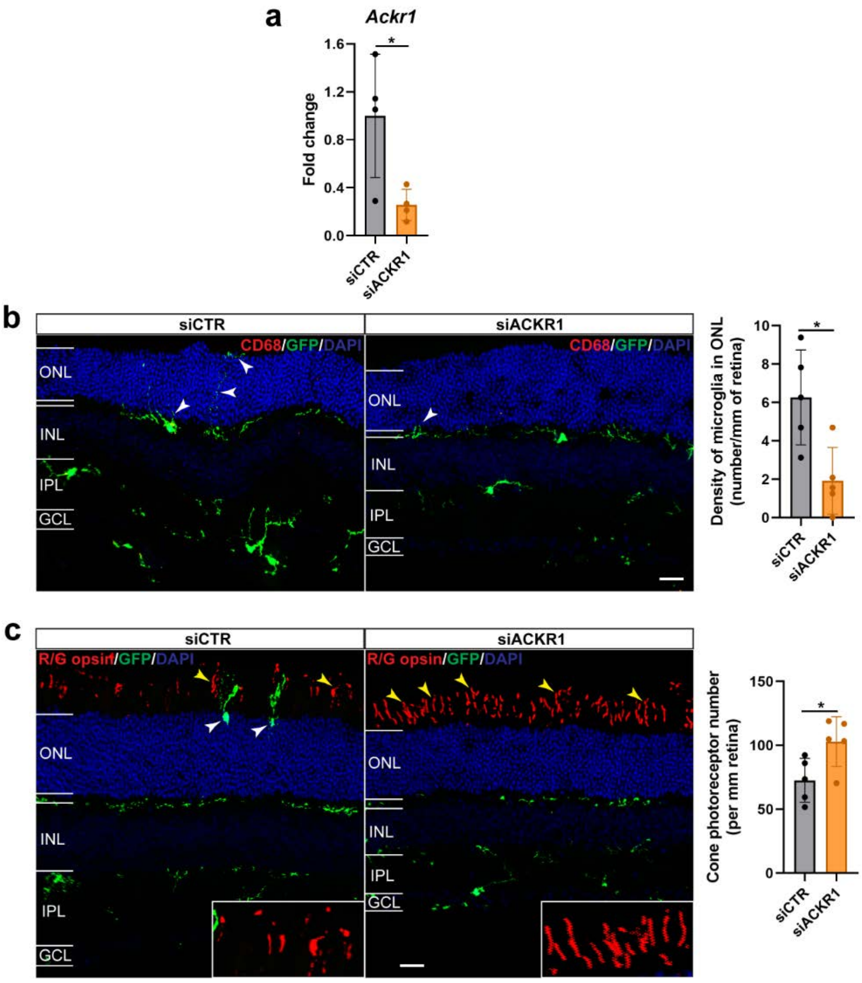

Supplementary Fig. 6: ACKR1 knockdown mitigates microglial activation and cone degeneration in CX3CR1-deficient retinas. a *Ackr1* mRNA levels in retinas of 6-week-old CX3CR1$^{GFP/GFP}$ mice following intravitreal injection of siACKR1 or siCTR, quantified by qPCR (n = 4 mice/group). **b** Representative immunofluorescence images of retinal sections stained with anti-CD68 antibody. White arrowheads indicate activated microglia extending processes into the ONL. Microglial density in the ONL was quantified (n = 5 mice/group). **c** Immunostaining of retinal sections with anti-R/G opsin antibody. Yellow arrowheads mark R/G opsin-positive cone photoreceptors. Boxed regions show high-magnification views of cone morphology. Quantification of surviving R/G opsin+ cones is shown (n = 5 mice/group). Scale bars: 20 µm. The data are presented as the means ± SEMs and were analysed via an unpaired two-tailed Student's *t* test (siACKR1 vs. siCTR, $*p < 0.05$).

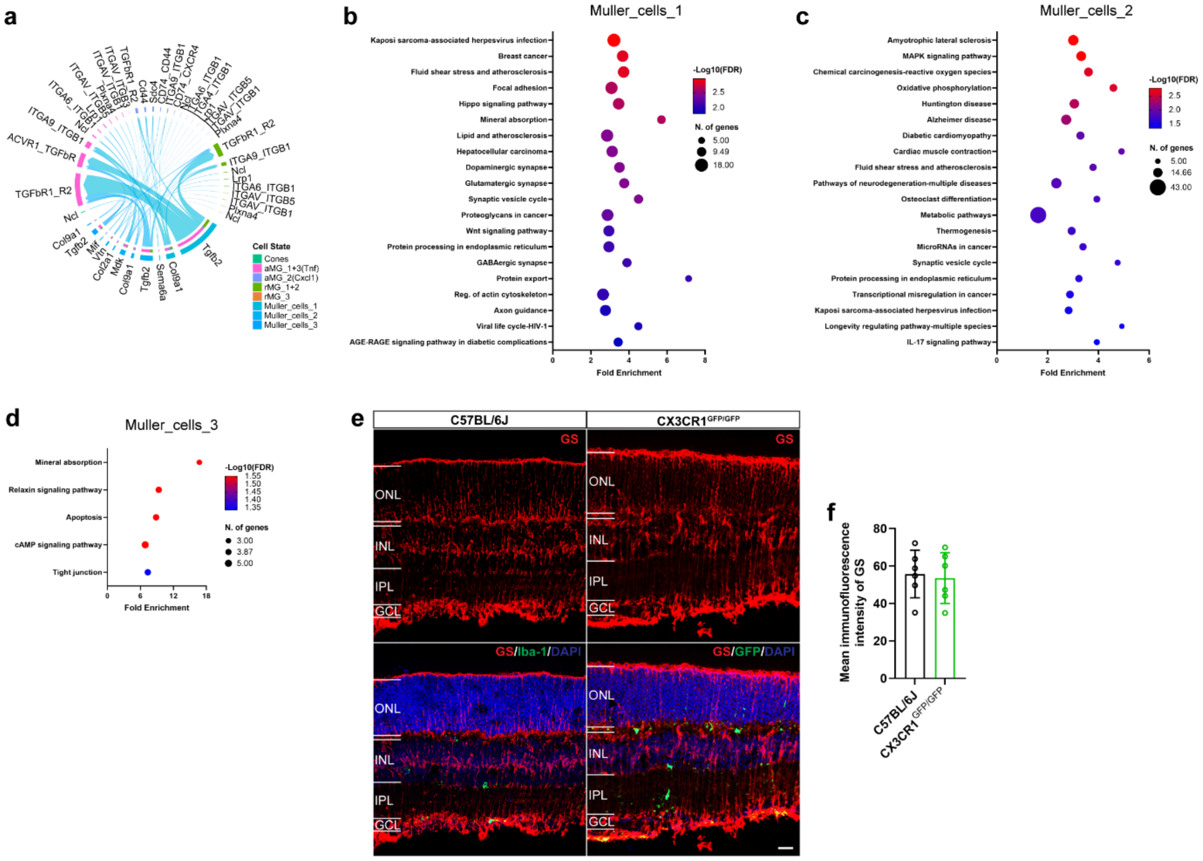

Supplementary Fig. 7: Müller cells were not involved in regulating cone photoreceptor degeneration in CX3CR1-deficient retinas. a Cell-cell communications among Müller cells, cones and microglia including aMG_1+3 (Tnf) and aMG_2 (Cxcl1) clusters. **b-d** KEGG pathway analysis of DEGs in Müller cells from CX3CR1$^{GFP/GFP}$ and C57BL/6J retinas at 6 weeks old. **e** Retinal sections stained with glutamine synthetase (GS) antibody from CX3CR1GFP/GFP and C57BL/6J mice at 6 weeks old. Scale bar, 20 μm. **f** Quantification of mean immunofluorescence intensity of GS in the retinal sections from CX3CR1$^{GFP/GFP}$ and C57BL/6J mice at 6 weeks old (n=6 mice/group). Data are presented as mean ± SEM and analysed by unpaired two-tailed Student's t test.

Supplementary Table 1: Sequences for primers and small interference RNAs.

Sequences for all primers

**Gene Name**	**Sequence (5'-3')**
Cx3cr1 (Forward)	TCGTCTTCACGTTCGGTCTG
Cx3cr1 (Reverse)	GTCAGTGATGCTCTTGGGCT
Stat3 (Forward)	CGAAGCCGACCCAGGTAGT
Stat3 (Reverse)	TATTGCTGCAGGTCGTTGGT
Tnfα (Forward)	ATGGCCTCCCTCTCATCAGT
Tnfα (Reverse)	TTTGCTACGACGTGGGCTAC
Il1β (Forward)	AATGCCACCTTTTGACAGTGATG
Il1β (Reverse)	GGAAGGTCCACGGGAAAGAC
Il6 (Forward)	GGGACTGATGCTGGTGACAA
Il6 (Reverse)	AGCATTGGAAATTGGGGTAGGA
Cox2 (Forward)	CTTCGGGAGCACAACAGAGT
Cox2 (Reverse)	AAGTGGTAACCGCTCAGGTG
Nos2 (Forward)	CCTCCTCCACCCTACCAAGT
Nos2 (Reverse)	CACCCAAAGTGCTTCAGTCA
Il10 (Forward)	GGCCCAGAAATCAAGGAGCA
Il10 (Reverse)	GCCTTGTAGACACCTTGGTCTT
Il13 (Forward)	CCTGGATTCCCTGACCAACA
Il13 (Reverse)	ATTTTGGTATCGGGGAGGCT
Bax (Forward)	GAACCATCATGGGCTGGACA
Bax (Reverse)	AGCCACCCTGGTCTTGGAT
Caspase-3 (Forward)	GGAGCAGCTTTGTGTGTGTG
Caspase-3 (Reverse)	AGCCTCCACCGGTATCTTCT
Bcl-2 (Forward)	AGTACCTGAACCGGCATCTG
Bcl-2 (Reverse)	GGTATGCACCCAGAGTGATG
C3 (Forward)	AGCTTCAGGGTCCCAGCTAC
C3 (Reverse)	GCTGGAATCTTGATGGAGACGC
H2-T23 (Forward)	GGACCGCGAATGACATAGC
H2-T23 (Reverse)	GCACCTCAGGGTGACTTCAT
Ggta1 (Forward)	GTGAACAGCATGAGGGGTTT
Ggta1 (Reverse)	GTTTTGTTGCCTCTGGGTGT
Iigp1 (Forward)	GGGGCAATAGCTCATTGGTA
Iigp1 (Reverse)	ACCTCGAAGACATCCCCTTT
Fbln5 (Forward)	CTTCAGATGCAAGCAACAA
Fbln5 (Reverse)	AGGCAGTGTCAGAGGCCTTA
Fkbp5 (Forward)	TATGCTTATGGCTCGGCTGG
Fkbp5 (Reverse)	CAGCCTTCCAGGTGGACTTT
Gbp2 (Forward)	GGGGTCACTGTCTGACCACT
Gbp2 (Reverse)	GGGAAACCTGGGATGAGATT
Clcf1 (Forward)	CTTCAATCCTCCTCGACTGG
Clcf1 (Reverse)	TACGTCGGAGTTCAGCTGTG

Cd109 (Forward)	CACAGTCGGGAGCCCTAAAG
Cd109 (Reverse)	GCAGCGATTTCGATGTCCAC
Ptgs2 (Forward)	GCTGTACAAGCAGTGGCAAA
Ptgs2 (Reverse)	CCCCAAAGATAGCATCTGGA
Cd14 (Forward)	GGACTGATCTCAGCCCTCTG
Cd14 (Reverse)	GCTTCAGCCCAGTGAAAGAC
S100a10 (Forward)	CCTCTGGCTGTGGACAAAAT
S100a10 (Reverse)	CTGCTCACAAGAAGCAGTGG
Bmp2 (Forward)	GGACCCGCTGTCTTCTAGTG
Bmp2 (Reverse)	GTCGAAGCTCTCCCACTGAC
Bmpr1b (Forward)	TTCTTCACCACGGAGGAAGC
Bmpr1b (Reverse)	TGGGGAATGAAGGCCGTAAC
Bmpr1a (Forward)	AGGATTCACCGAAAGCCCAG
Bmpr1a (Reverse)	ACGCATTAACACCGTCTGGT
Ackr1 (Forward)	TACAATGGCTTCTGCGCCTT
Ackr1 (Reverse)	GTGCAGCATACTCAGGGCTT

Sequences for small interference RNAs

**Oligonucleotides Resource**	**Sequence (5'-3')**
mm-Bmpr1b-si-1 (Sense)	GCACAGAUGGGUACUGCUUTT
mm-Bmpr1b-si-1 (Anti-sense)	AAGCAGUACCCAUCUGUGCTT
mm-Bmpr1b-si-2 (Sense)	GCAGGACGAGACAUACAUUTT
mm-Bmpr1b-si-2 (Anti-sense)	AAUGUAUGUCUCGUCCUGCTT
mm-Bmpr1b-si-3 (Sense)	CCACCACCUUAGACGCAAATT
mm-Bmpr1b-si-3 (Anti-sense)	UUUGCGUCUAAGGUGGUGGTT
mm-Bmpr1a-si-1 (Sense)	CAUCAUUUCUCAUGUUCAAGGTT
mm-Bmpr1a-si-1 (Anti-sense)	CCUUGAACAUGAGAAAUGAUGTT
mm-Bmpr1a-si-2 (Sense)	GCUACGCAGGACAAUAGAAUGTT
mm-Bmpr1a-si-2 (Anti-sense)	CAUUCUAUUGUCCUGCGUAGCTT
mm-Bmpr1a-si-3 (Sense)	GAAGAAGCUAGCUGGUUUAGATT
mm-Bmpr1a-si-3 (Anti-sense)	UCUAAACCAGCUAGCUUCUUCTT
mm-Ackr1-si-1 (Sense)	GCCCAUUCUGGCAGAGUUATT
mm-Ackr1-si-1 (Anti-sense)	UAACUCUGCCAGAAUGGGCTT
mm-Ackr1-si-2 (Sense)	CAGACUGAAUAUUGGUCAACUTT
mm-Ackr1-si-2 (Anti-sense)	AGUUGACCAAUAUUCAGUCUGTT
mm-Ackr1-si-3 (Sense)	GAGACAUGGAAGCUUUGAAGUTT
mm-Ackr1-si-3 (Anti-sense)	ACUUCAAAGCUUCCAUGUCUCTT
